# Supplementary material for: Measuring real-time disease transmissibility with temperature-dependent generation intervals
Source: PLoS Comput Biol. 2026 Jan 21;22(1):e1013820. doi: 10.1371/journal.pcbi.1013820 (PMC12854435; doi:10.1371/journal.pcbi.1013820)
Supplement: S1 File — (DOCX) [file pcbi.1013820.s001.docx]

**Supplementary Information for “Measuring real-time disease transmissibility with temperature-dependent generation intervals”**

Esther Li Wen Choo^1^, Kris V Parag^2^, Jo Yi Chow^1^, Jue Tao Lim^1, #^

^1^Lee Kong Chian School of Medicine, Nanyang Technological University, Singapore

^2^MRC Centre for Global Infectious Disease Analysis, Imperial College London, London, United Kingdom

^#^Corresponding Author: Lim Jue Tao, juetao.lim@ntu.edu.sg

Table of Contents

[Fig A in S1 File. True underlying R_t_ used for generating dengue case counts. 3](#_Toc216209343)

[Fig B in S1 File. Temperature datasets used to define temperature-dependent generation interval in simulation. 4](#_Toc216209344)

[Fig C in S1 File. AUC-ROC curves based on estimated reproduction numbers in simulation. 5](#_Toc216209345)

[Fig D in S1 File. Temperature and underlying R_t_ used for simulation and respective estimated R_t_ values. 17](#_Toc216209346)

[Fig E in S1 File. Mean of generation time distribution over days. Temperature-dependent generation intervals were based on daily temperature in Singapore from 2012 to 2024. 21](#_Toc216209347)

[Fig F in S1 File. 1-step ahead dengue predictions by reproduction numbers. 22](#_Toc216209348)

[Fig G in S1 File. Real-time transmissibility estimates with simulated reporting delays. 23](#_Toc216209349)

[Text A in S1 File. Derivation of angular reproduction number. 23](#_Toc216209350)

[Table A in S1 File. Percentage accuracy of estimated angular reproduction number (Omega), temperature-independent reproduction number (ti-R_t_) and temperature-dependent reproduction number (td-R_t_) under simulations when transmission rate was overestimated by 4 times. 24](#_Toc216209351)

[Table B in S1 File. Percentage accuracy of estimated angular reproduction number (Omega), temperature-independent reproduction number (ti-R_t_) and temperature-dependent reproduction number (td-R_t_) under simulations when transmission rate was overestimated by 3 times. 25](#_Toc216209352)

[Table C in S1 File. Percentage accuracy of estimated angular reproduction number (Omega), temperature-independent reproduction number (ti-R_t_) and temperature-dependent reproduction number (td-R_t_) under simulations when transmission rate was underestimated by 3 times. 26](#_Toc216209353)

[Table D in S1 File. Percentage accuracy of estimated angular reproduction number (Omega), temperature-independent reproduction number (ti-R_t_) and temperature-dependent reproduction number (td-R_t_) under simulations when transmission rate was underestimated by 4 times. 27](#_Toc216209354)

[Table E in S1 File. AUC-ROC values based on estimated reproduction numbers in simulation when transmission rate is overestimated and underestimated by 3 or 4 times respectively . 28](#_Toc216209355)

Fig A in S1 File. True underlying R_t_ used for generating dengue case counts. 18 sets of R_t_ were pre-determined using a sine function, with varying amplitudes (ampli) and periods to test the robustness of the reproduction number estimation. Random noise was also added to half of the R_t_ values. Dengue case counts were generated through the renewal model using the pre-determined R_t_ values, with 1 set of dengue case counts corresponding to each set of R_t_ values.

Fig B in S1 File. Temperature datasets used to define temperature-dependent generation interval in simulation. Each dataset was used in separate simulation sets to be incorporated into the generation interval distribution, which was subsequently used to generate synthetic dengue case counts through the renewal model.


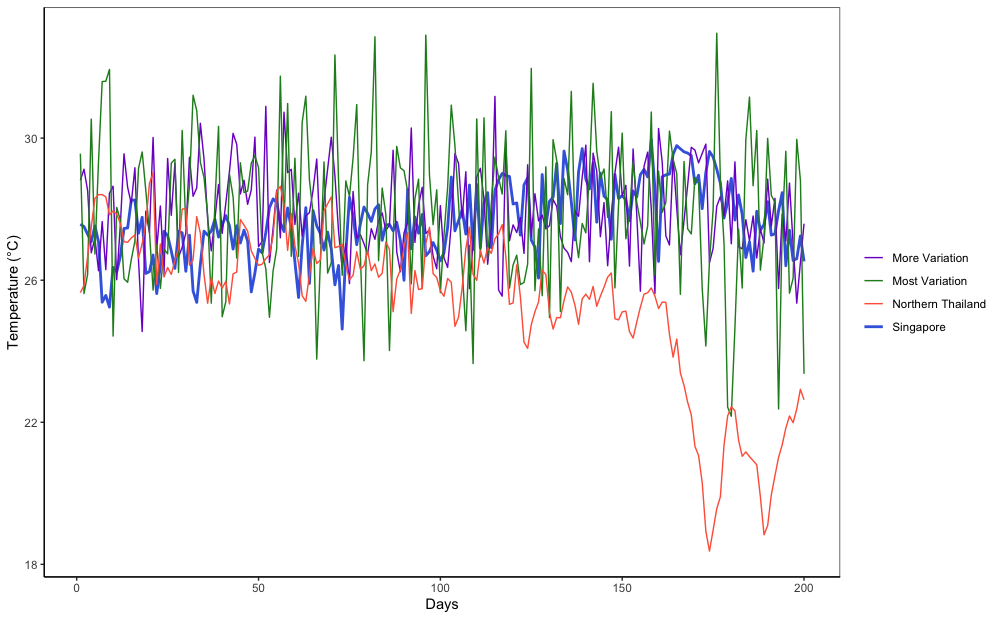


# Fig C **in S1 File**. AUC-ROC curves based on estimated reproduction numbers in simulation.

Each curve shows the ability of the temperature-independent reproduction number (ti-Rt), angular reproduction number (omega) and temperature-dependent reproduction number (td-Rt) to classify periods of transmission risk, where R_t_ > 1 is treated as the positive class (indicating epidemic growth) and R_t_$\leq$1 as the negative class. Higher AUC-ROC values indicate better classification performance. AUC-ROC values are labelled in each plot. Each row corresponds to 1 simulation scenario with its respective temperature dataset and R_t_ dynamics.


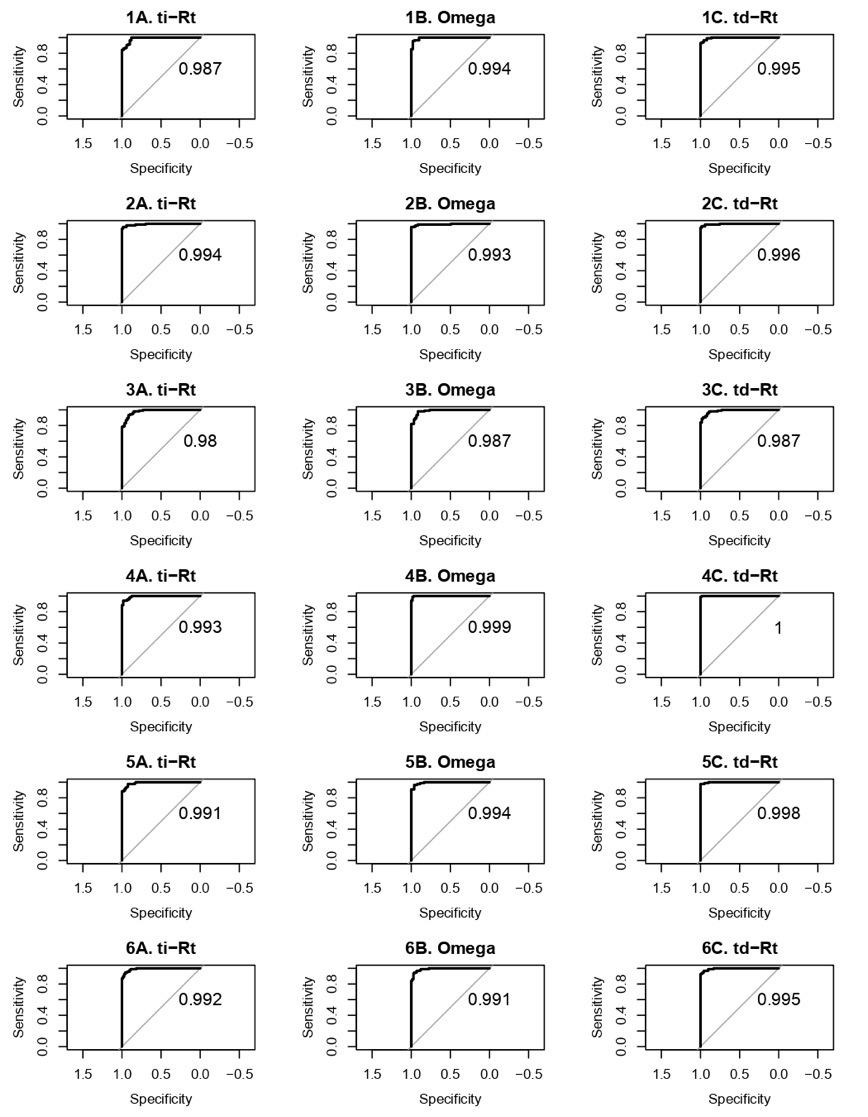


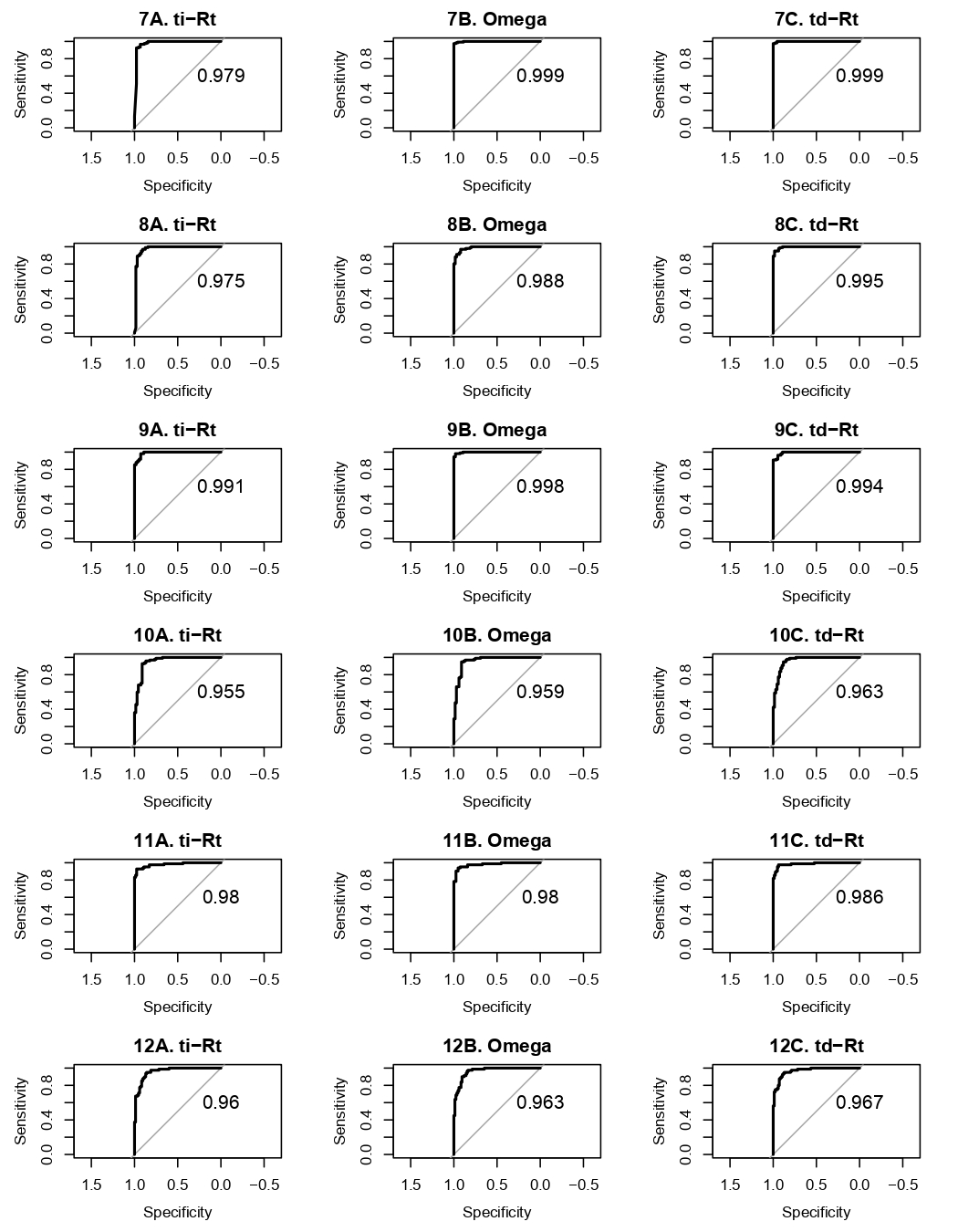

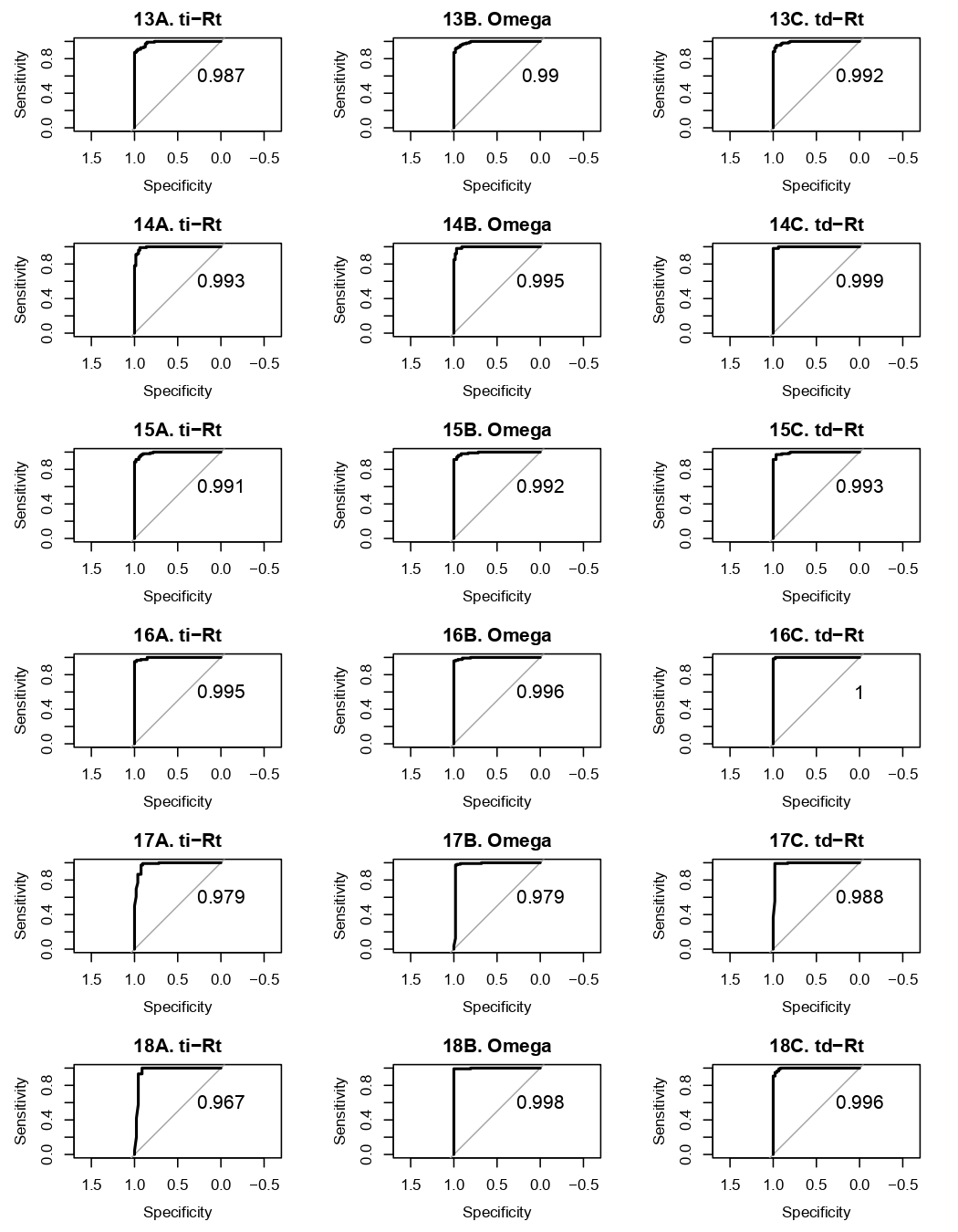

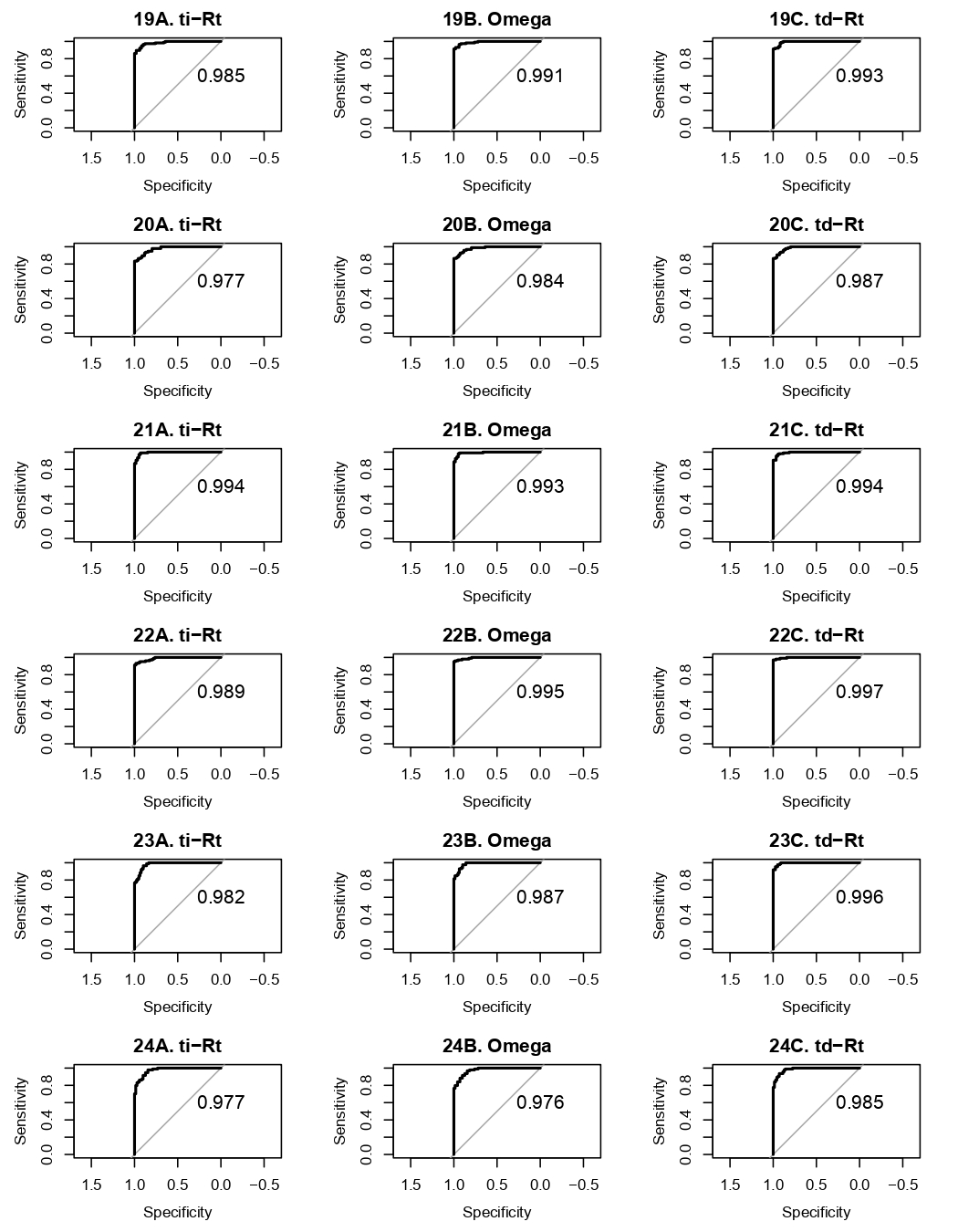

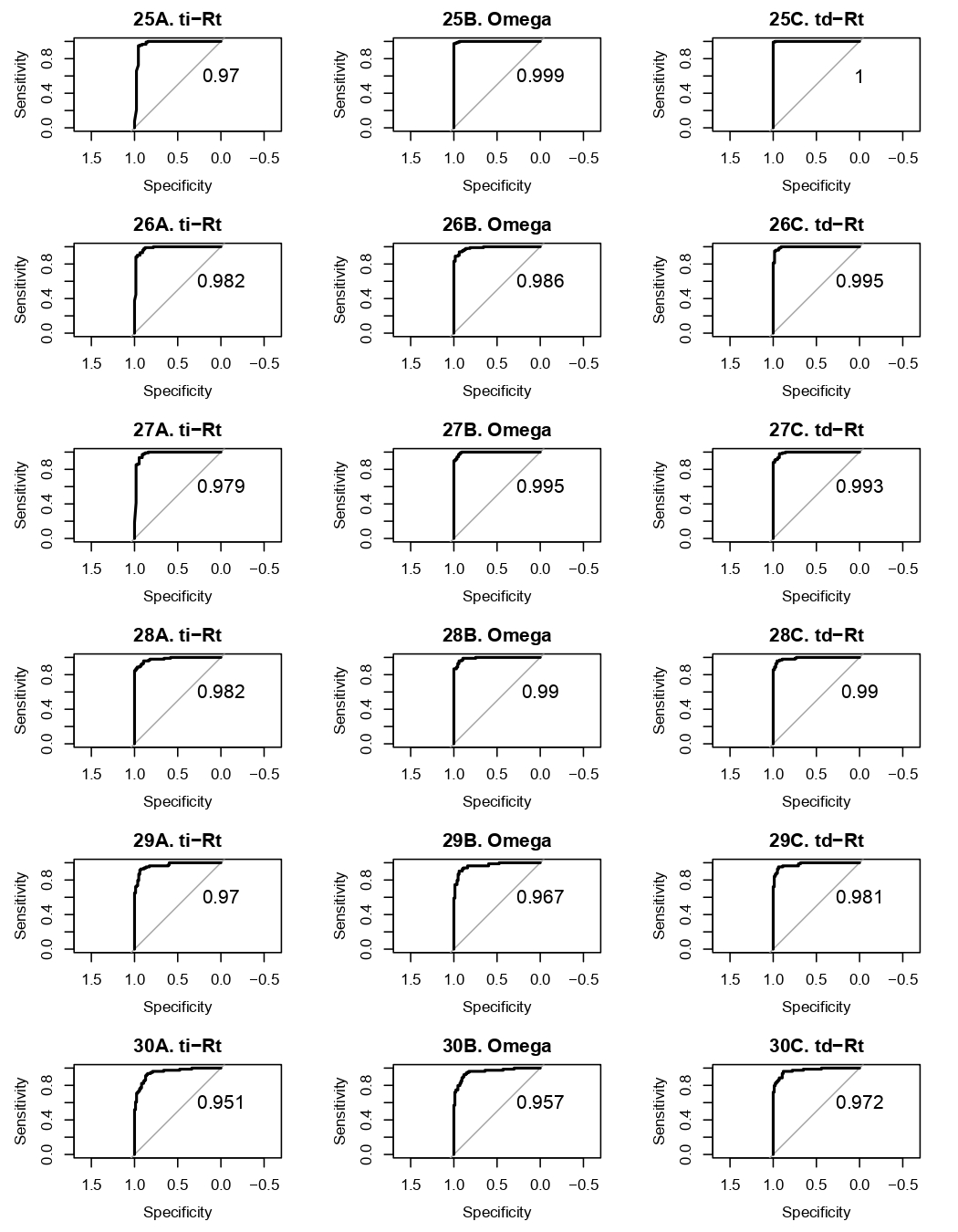

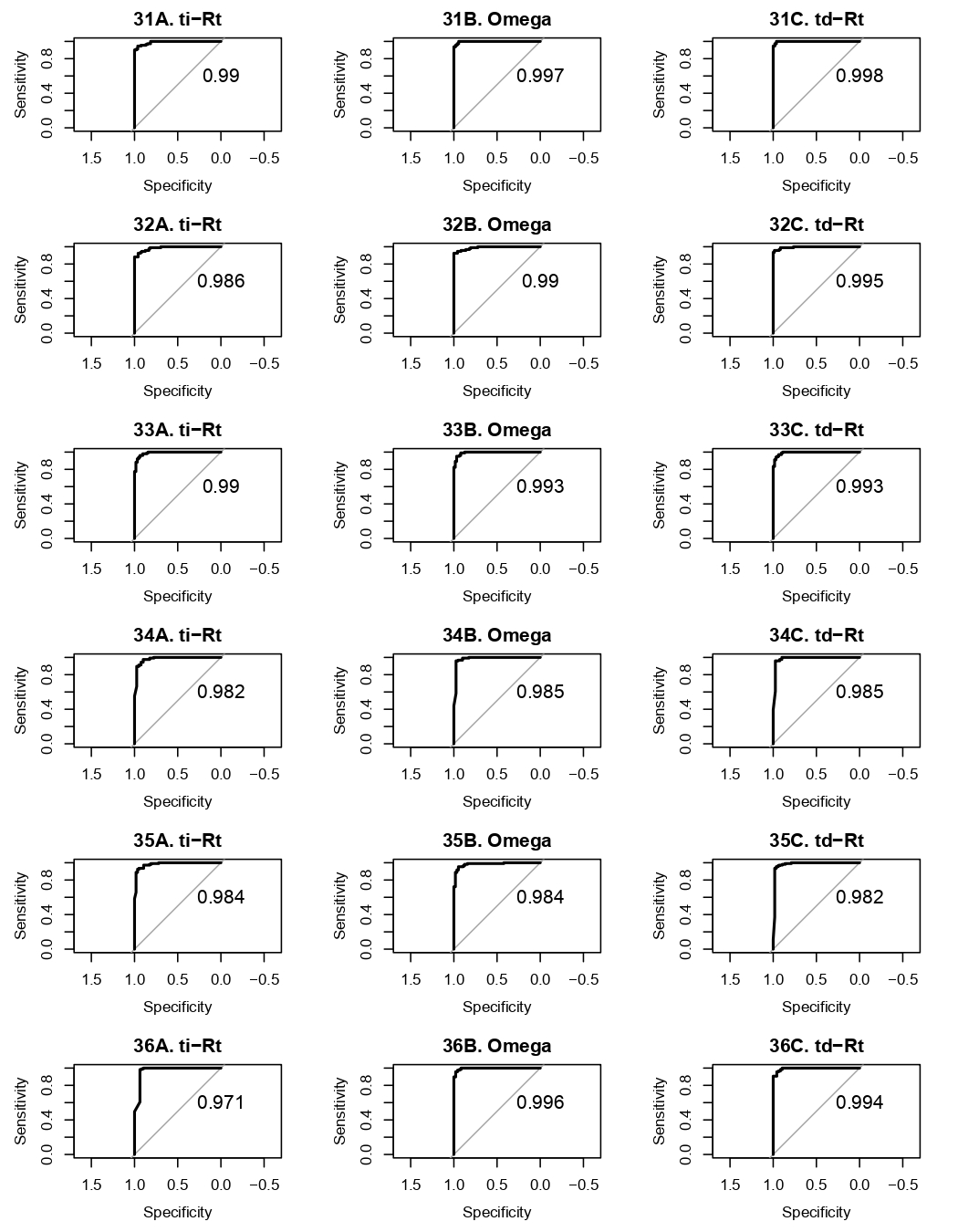

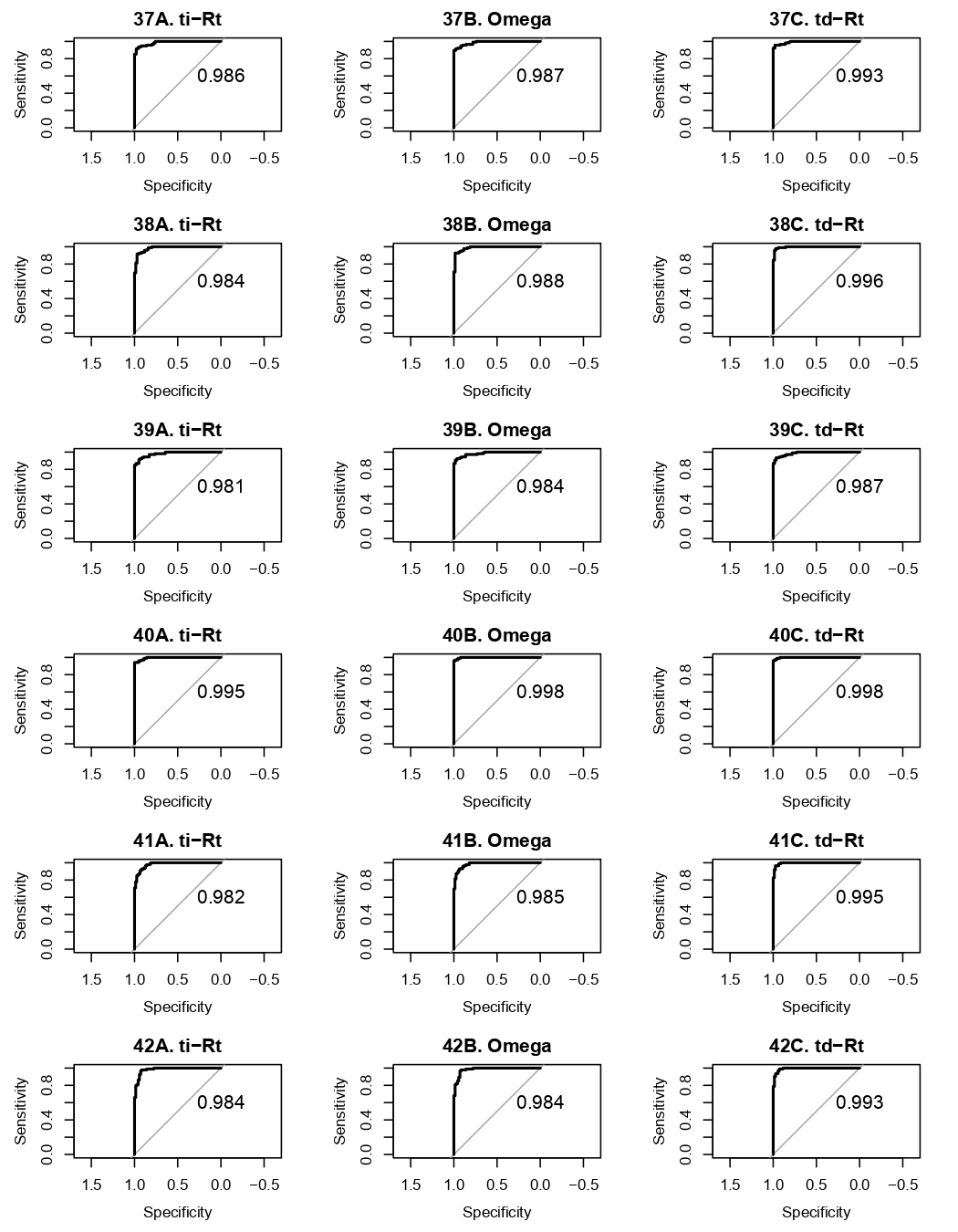

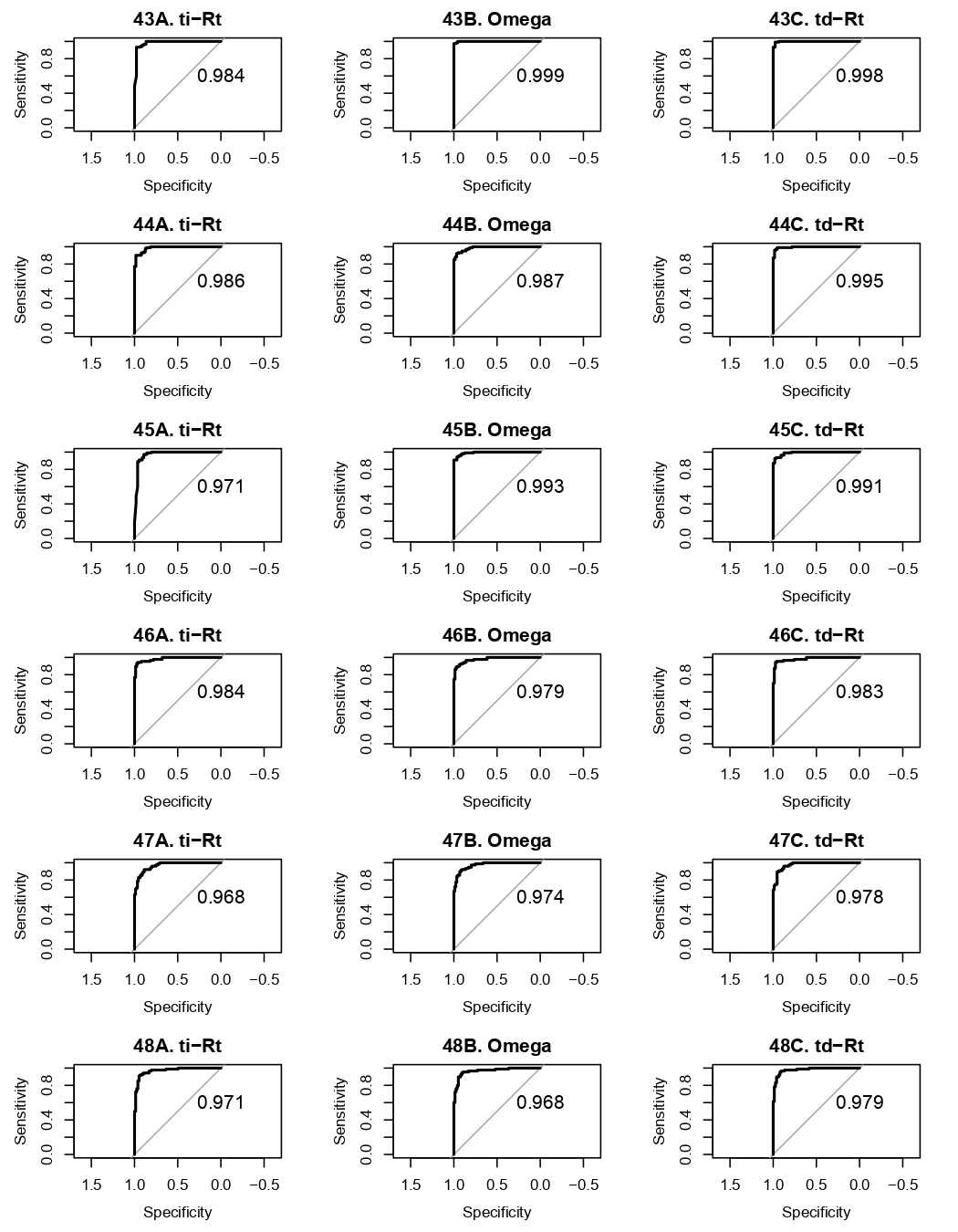

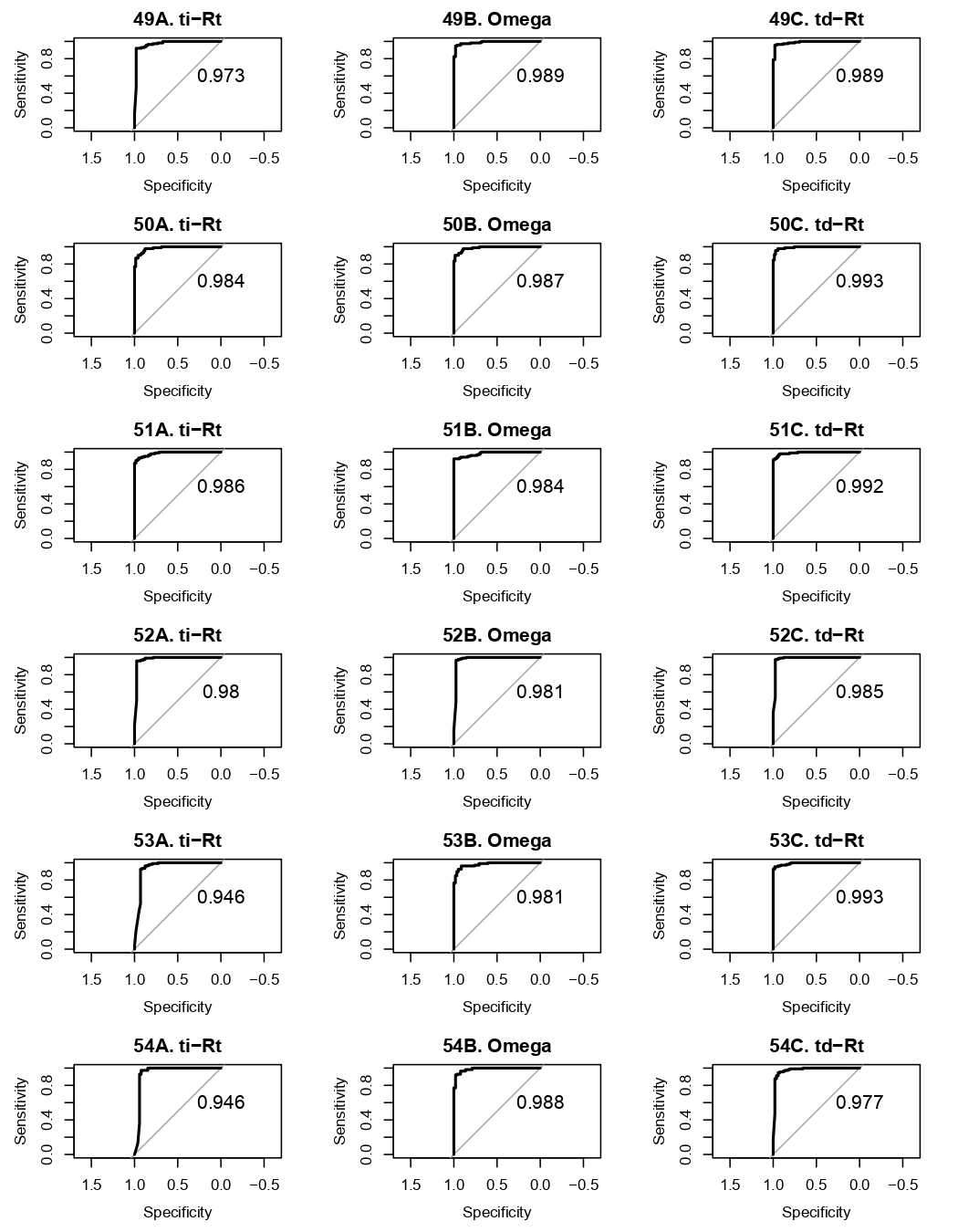

# Fig D in S1 File. Temperature and underlying R_t_ used for simulation and respective estimated R_t_ values.

The angular reproduction number (Omega), estimated temperature-independent reproduction number (ti-Rt) and estimated temperature-dependent reproduction number (td-Rt) were estimated on simulated dengue data, where each row represents a single simulation scenario and the data used respectively. The values in the td-Rt, ti-Rt and Omega columns represent the percentage agreement by the threshold of 1 with the true Rt. The red line represents true values while the black line represents estimated values.

# Fig E in S1 File. Mean of generation time distribution over days. Temperature-dependent generation intervals were based on daily temperature in Singapore from 2012 to 2024.


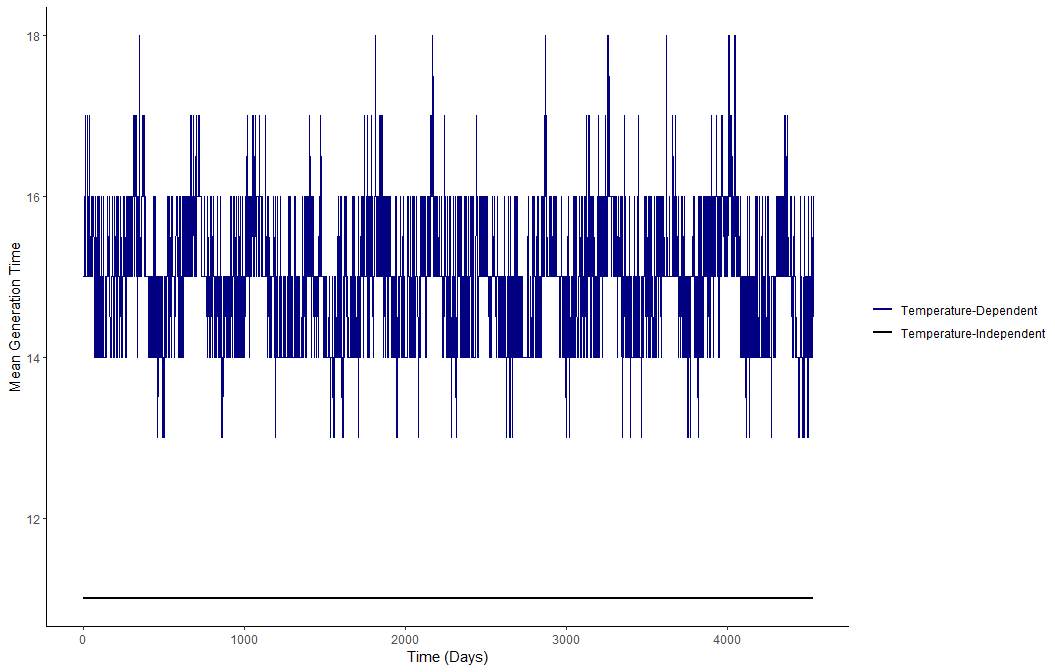


Fig F in S1 File. 1-step ahead dengue predictions by reproduction numbers. The temperature-dependent reproduction number, temperature-independent reproduction number and angular reproduction number was estimated using the EpiFilter algorithm, which also outputs the posterior predictive distribution for the respective reproduction number. The 1-step ahead prediction was obtained from the posterior mean of the predictive distribution and the 95% credible intervals were obtained from the 2.5% and 97.5% quantile of the posterior predictive distribution. The mean absolute percentage error (MAPE) of each set of predictions was calculated and labelled below.


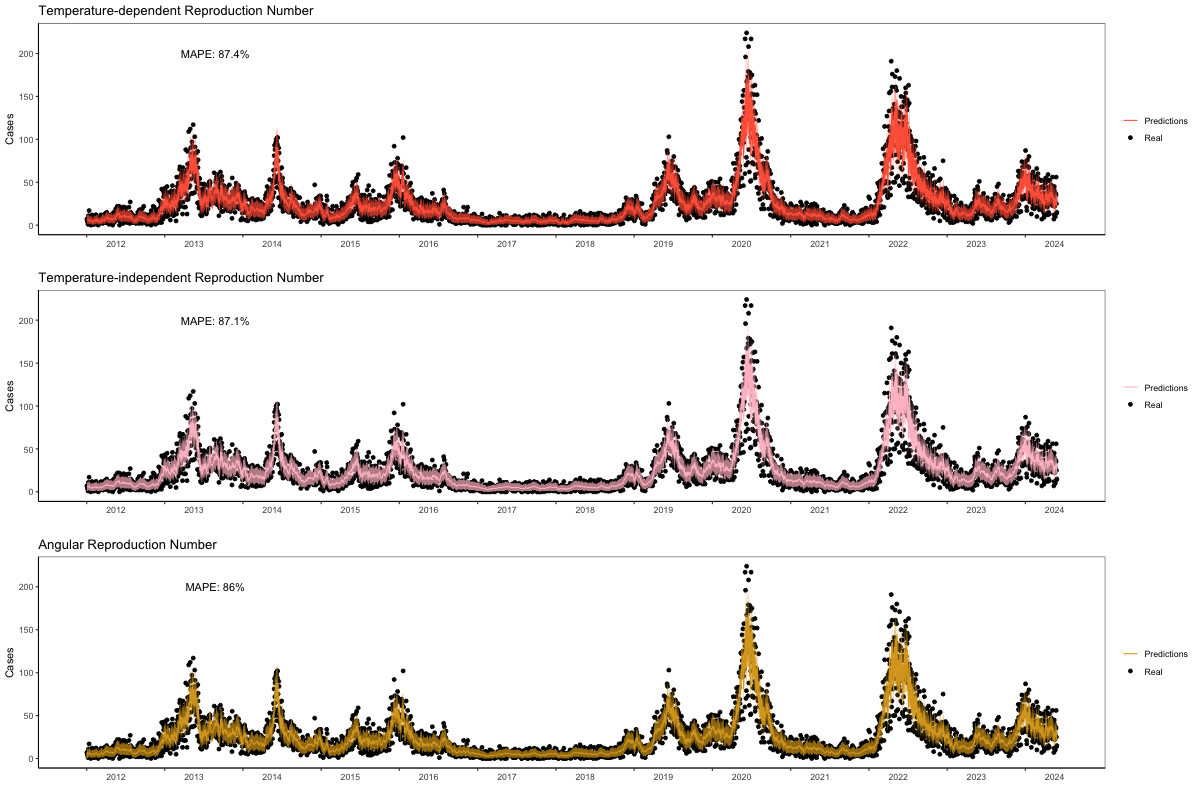


Fig G in S1 File. Real-time transmissibility estimates with simulated reporting delays.

Each line shows the daily real-time estimates of temperature-dependent reproduction number (td-R_t_), temperature-independent reproduction number (ti-R_t_) and angular reproduction number (Omega) obtained using only data available up to that day, incorporating a lognormal reporting delay (mean = 3 days, SD = 3 days). The estimates capture how underreporting and right truncation influence transmissibility estimates over time, with all three metrics showing similar temporal patterns.


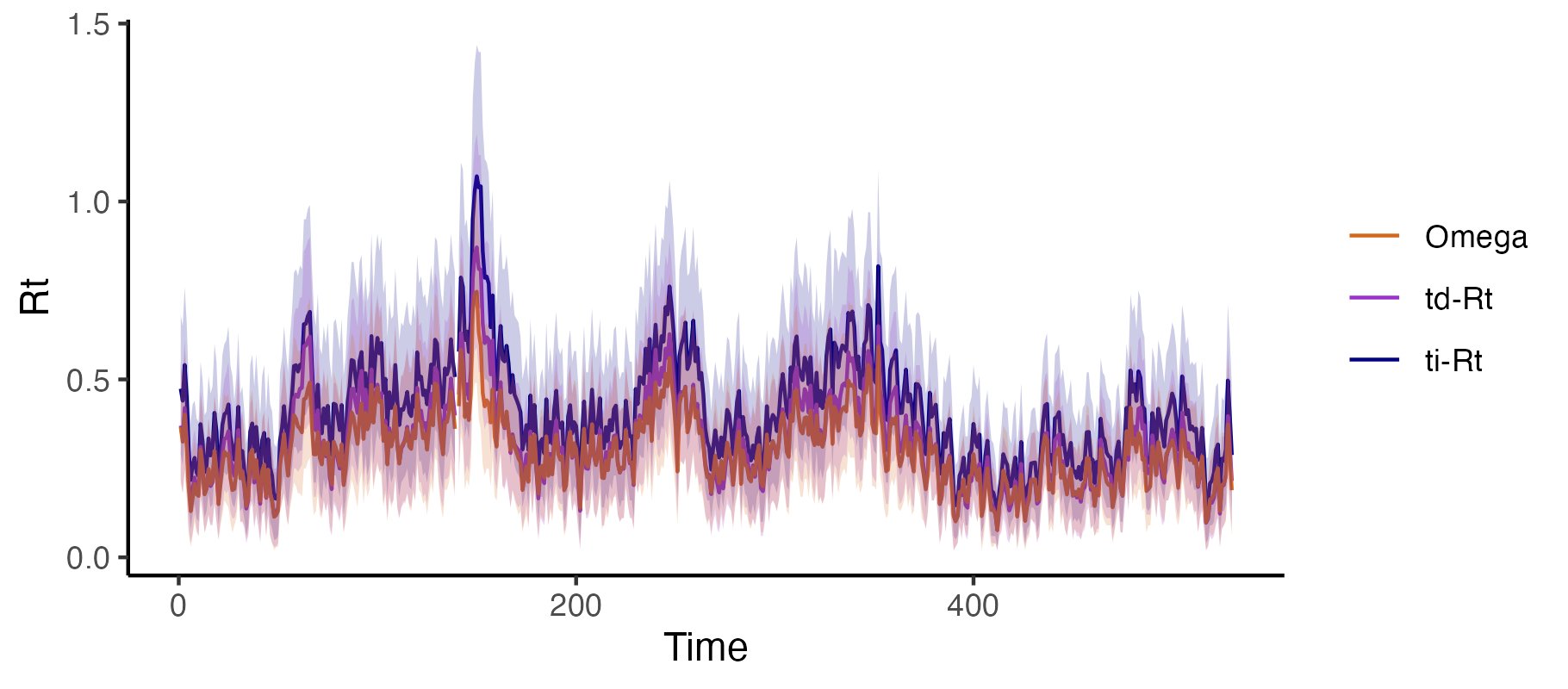


# Text A in S1 File. Derivation of angular reproduction number.

Total infectiousness, $\Lambda_{t}$, can be interpreted as a dot product between the vectors of generation time probabilities $w:=w_{1}^{m}$ and past incidence $I:=I_{t-m}^{t-1}$ over the support of the generation time distribution, $m$:

$$\Lambda_{t}=\left\| w \right\|\left\| I \right\|cos\theta_{t} =\frac{\left\| w \right\|}{\left\| w_{max} \right\|}M_{t}cos\theta_{t}$$

where $\left\| X \right\|$ is the Euclidean norm of X and $\theta_{t}$ is the time varying angle between $w$ and $I$. We can compute the root mean square of the incidence across the support of the generation time distribution as $M_{t}:=\frac{1}{\sqrt{m}}\left\| I \right\|$. Furthermore, under the constraint that $\sum_{u=1}^{m} w_{u}=1$, the maximum value of the generation time is $\left\| w_{max} \right\|=\frac{1}{\sqrt{m}}$, giving rise to the right expression in the equation above. Using the new expression for $\Lambda_{t}$, the expected cases of incidence can be re-defined:

$$E[I_{t}]=R_{t}\Lambda_{t}=\left( \frac{\left\| w \right\|}{\left\| w_{max} \right\|}R_{t} cos\theta_{t} \right)M_{t}$$

This gives a new metric, $\Omega_{t}$, that only requires expected incidence, $E[I_{t}]$, and the root mean square of past incidence, $M_{t}$:

$$\Omega_{t}=\frac{\left\| w \right\|}{\left\| w_{max} \right\|}R_{t} cos\theta_{t}=E[I_{t}]M_{t}^{-1}$$

# Table A in S1 File. Percentage accuracy of estimated angular reproduction number (Omega), temperature-independent reproduction number (ti-R_t_) and temperature-dependent reproduction number (td-R_t_) under simulations when transmission rate was overestimated by 4 times.

Percentage accuracy was calculated based on whether the significant transmissibility estimates and true Rt were both below 1 or above 1. The transmissibility estimate with the highest accuracy in each scenario was bolded.

| Noisy R |  |  |  |  |  |  | Smooth R |  |  |  |  |  |
| --- | --- | --- | --- | --- | --- | --- | --- | --- | --- | --- | --- | --- |
| Period | Amplitude | Temperature | Omega | td-Rt | ti-Rt |  | Period | Amplitude | Temperature | Omega | td-Rt | ti-Rt |
| 0.05 | 1 | Singapore data | 0.318 | 0.453 | **0.653** |  | 0.05 | 1 | Singapore data | 0.347 | 0.582 | **0.735** |
| 0.1 | 1 | Singapore data | **0.459** | 0.447 | 0.324 |  | 0.1 | 1 | Singapore data | 0.388 | **0.565** | 0.441 |
| 0.15 | 1 | Singapore data | 0.365 | **0.476** | 0.441 |  | 0.15 | 1 | Singapore data | 0.353 | **0.482** | 0.471 |
| 0.05 | 2 | Singapore data | 0.776 | 0.812 | **0.865** |  | 0.05 | 2 | Singapore data | 0.483 | 0.782 | **0.818** |
| 0.1 | 2 | Singapore data | 0.771 | 0.835 | **0.847** |  | 0.1 | 2 | Singapore data | 0.582 | **0.8** | 0.782 |
| 0.15 | 2 | Singapore data | 0.7 | 0.806 | **0.818** |  | 0.15 | 2 | Singapore data | 0.518 | 0.776 | **0.782** |
| 0.05 | 3 | Singapore data | 0.906 | 0.918 | **0.924** |  | 0.05 | 3 | Singapore data | 0.906 | 0.906 | **0.935** |
| 0.1 | 3 | Singapore data | 0.859 | 0.906 | **0.912** |  | 0.1 | 3 | Singapore data | 0.859 | **0.912** | 0.888 |
| 0.15 | 3 | Singapore data | 0.882 | 0.9 | **0.912** |  | 0.15 | 3 | Singapore data | 0.882 | **0.924** | 0.918 |
| 0.05 | 1 | More Variance | 0.5 | **0.635** | 0.582 |  | 0.05 | 1 | More Variance | 0.471 | 0.576 | **0.641** |
| 0.1 | 1 | More Variance | 0.376 | 0.347 | **0.388** |  | 0.1 | 1 | More Variance | 0.512 | **0.594** | 0.535 |
| 0.15 | 1 | More Variance | 0.418 | **0.565** | 0.441 |  | 0.15 | 1 | More Variance | 0.5 | **0.665** | 0.594 |
| 0.05 | 2 | More Variance | 0.747 | 0.782 | **0.812** |  | 0.05 | 2 | More Variance | 0.706 | 0.782 | **0.824** |
| 0.1 | 2 | More Variance | 0.765 | **0.829** | 0.818 |  | 0.1 | 2 | More Variance | 0.635 | **0.782** | **0.782** |
| 0.15 | 2 | More Variance | 0.8 | **0.853** | **0.853** |  | 0.15 | 2 | More Variance | 0.547 | 0.759 | **0.776** |
| 0.05 | 3 | More Variance | 0.882 | 0.906 | **0.941** |  | 0.05 | 3 | More Variance | 0.912 | 0.918 | **0.924** |
| 0.1 | 3 | More Variance | 0.859 | **0.912** | **0.912** |  | 0.1 | 3 | More Variance | 0.876 | **0.912** | 0.9 |
| 0.15 | 3 | More Variance | 0.906 | 0.912 | **0.935** |  | 0.15 | 3 | More Variance | 0.871 | **0.906** | **0.906** |
| 0.05 | 1 | Most Variance | 0.371 | 0.535 | **0.735** |  | 0.05 | 1 | Most Variance | 0.435 | 0.559 | **0.676** |
| 0.1 | 1 | Most Variance | 0.429 | **0.518** | 0.453 |  | 0.1 | 1 | Most Variance | 0.406 | **0.582** | 0.559 |
| 0.15 | 1 | Most Variance | **0.447** | 0.329 | 0.176 |  | 0.15 | 1 | Most Variance | 0.406 | **0.606** | 0.541 |
| 0.05 | 2 | Most Variance | 0.788 | 0.806 | **0.841** |  | 0.05 | 2 | Most Variance | 0.629 | 0.759 | **0.806** |
| 0.1 | 2 | Most Variance | 0.782 | 0.824 | **0.835** |  | 0.1 | 2 | Most Variance | 0.565 | **0.771** | 0.724 |
| 0.15 | 2 | Most Variance | 0.806 | **0.859** | **0.859** |  | 0.15 | 2 | Most Variance | 0.647 | 0.788 | **0.812** |
| 0.05 | 3 | Most Variance | 0.906 | 0.906 | **0.935** |  | 0.05 | 3 | Most Variance | 0.894 | 0.894 | **0.906** |
| 0.1 | 3 | Most Variance | 0.882 | **0.929** | 0.912 |  | 0.1 | 3 | Most Variance | 0.871 | **0.924** | 0.918 |
| 0.15 | 3 | Most Variance | 0.882 | 0.906 | **0.912** |  | 0.15 | 3 | Most Variance | 0.894 | **0.929** | **0.929** |

# Table B in S1 File. Percentage accuracy of estimated angular reproduction number (Omega), temperature-independent reproduction number (ti-R_t_) and temperature-dependent reproduction number (td-R_t_) under simulations when transmission rate was overestimated by 3 times.

Percentage accuracy was calculated based on whether the significant transmissibility estimates and true Rt were both below 1 or above 1. The transmissibility estimate with the highest accuracy in each scenario was bolded.

| Noisy R |  |  |  |  |  |  | Smooth R |  |  |  |  |  |
| --- | --- | --- | --- | --- | --- | --- | --- | --- | --- | --- | --- | --- |
| Period | Amplitude | Temperature | Omega | td-Rt | ti-Rt |  | Period | Amplitude | Temperature | Omega | td-Rt | ti-Rt |
| 0.05 | 1 | Singapore data | 0.371 | 0.418 | **0.588** |  | 0.05 | 1 | Singapore data | 0.576 | **0.759** | 0.706 |
| 0.1 | 1 | Singapore data | **0.441** | 0.388 | 0.324 |  | 0.1 | 1 | Singapore data | 0.476 | **0.606** | 0.471 |
| 0.15 | 1 | Singapore data | 0.382 | **0.453** | 0.447 |  | 0.15 | 1 | Singapore data | 0.523 | **0.635** | 0.582 |
| 0.05 | 2 | Singapore data | 0.8 | 0.818 | **0.835** |  | 0.05 | 2 | Singapore data | 0.759 | 0.794 | **0.835** |
| 0.1 | 2 | Singapore data | 0.8 | **0.818** | 0.782 |  | 0.1 | 2 | Singapore data | 0.747 | **0.824** | 0.812 |
| 0.15 | 2 | Singapore data | 0.818 | 0.876 | **0.865** |  | 0.15 | 2 | Singapore data | 0.635 | 0.818 | **0.824** |
| 0.05 | 3 | Singapore data | 0.894 | 0.894 | **0.924** |  | 0.05 | 3 | Singapore data | 0.924 | **0.935** | 0.929 |
| 0.1 | 3 | Singapore data | 0.894 | **0.924** | 0.882 |  | 0.1 | 3 | Singapore data | 0.888 | **0.929** | 0.918 |
| 0.15 | 3 | Singapore data | 0.894 | 0.912 | **0.918** |  | 0.15 | 3 | Singapore data | 0.894 | **0.941** | 0.929 |
| 0.05 | 1 | More Variance | 0.324 | 0.424 | **0.594** |  | 0.05 | 1 | More Variance | 0.547 | 0.653 | **0.682** |
| 0.1 | 1 | More Variance | 0.441 | **0.576** | 0.5 |  | 0.1 | 1 | More Variance | 0.429 | **0.571** | 0.535 |
| 0.15 | 1 | More Variance | 0.412 | **0.582** | 0.529 |  | 0.15 | 1 | More Variance | 0.547 | **0.706** | 0.606 |
| 0.05 | 2 | More Variance | 0.759 | 0.776 | **0.841** |  | 0.05 | 2 | More Variance | 0.818 | 0.853 | **0.859** |
| 0.1 | 2 | More Variance | 0.782 | **0.841** | 0.8 |  | 0.1 | 2 | More Variance | 0.753 | **0.794** | 0.776 |
| 0.15 | 2 | More Variance | 0.859 | **0.888** | 0.882 |  | 0.15 | 2 | More Variance | 0.559 | 0.818 | **0.835** |
| 0.05 | 3 | More Variance | **0.929** | **0.929** | **0.929** |  | 0.05 | 3 | More Variance | 0.918 | **0.924** | **0.924** |
| 0.1 | 3 | More Variance | 0.9 | **0.929** | 0.882 |  | 0.1 | 3 | More Variance | 0.865 | **0.918** | 0.906 |
| 0.15 | 3 | More Variance | 0.912 | **0.941** | 0.935 |  | 0.15 | 3 | More Variance | 0.912 | **0.935** | 0.918 |
| 0.05 | 1 | Most Variance | 0.359 | 0.488 | **0.641** |  | 0.05 | 1 | Most Variance | 0.588 | 0.671 | **0.729** |
| 0.1 | 1 | Most Variance | 0.435 | **0.612** | 0.535 |  | 0.1 | 1 | Most Variance | 0.494 | 0.576 | **0.606** |
| 0.15 | 1 | Most Variance | 0.359 | 0.382 | **0.412** |  | 0.15 | 1 | Most Variance | 0.553 | **0.718** | 0.606 |
| 0.05 | 2 | Most Variance | 0.853 | 0.835 | **0.847** |  | 0.05 | 2 | Most Variance | 0.747 | 0.788 | **0.818** |
| 0.1 | 2 | Most Variance | 0.747 | **0.853** | 0.835 |  | 0.1 | 2 | Most Variance | 0.671 | **0.835** | **0.835** |
| 0.15 | 2 | Most Variance | 0.8 | **0.847** | 0.841 |  | 0.15 | 2 | Most Variance | 0.724 | 0.818 | **0.824** |
| 0.05 | 3 | Most Variance | 0.912 | 0.929 | **0.941** |  | 0.05 | 3 | Most Variance | 0.9 | **0.929** | 0.924 |
| 0.1 | 3 | Most Variance | 0.929 | **0.941** | 0.906 |  | 0.1 | 3 | Most Variance | 0.888 | **0.929** | 0.912 |
| 0.15 | 3 | Most Variance | 0.888 | 0.9 | **0.912** |  | 0.15 | 3 | Most Variance | 0.912 | **0.935** | 0.918 |

# Table C in S1 File. Percentage accuracy of estimated angular reproduction number (Omega), temperature-independent reproduction number (ti-R_t_) and temperature-dependent reproduction number (td-R_t_) under simulations when transmission rate was underestimated by 3 times.

Percentage accuracy was calculated based on whether the significant transmissibility estimates and true Rt were both below 1 or above 1. The transmissibility estimate with the highest accuracy in each scenario was bolded.

| Noisy R |  |  |  |  |  |  | Smooth R |  |  |  |  |  |
| --- | --- | --- | --- | --- | --- | --- | --- | --- | --- | --- | --- | --- |
| Period | Amplitude | Temperature | Omega | td-Rt | ti-Rt |  | Period | Amplitude | Temperature | Omega | td-Rt | ti-Rt |
| 0.05 | 1 | Singapore data | 0.588 | 0.653 | **0.712** |  | 0.05 | 1 | Singapore data | 0.718 | **0.782** | 0.735 |
| 0.1 | 1 | Singapore data | 0.541 | **0.682** | 0.6 |  | 0.1 | 1 | Singapore data | 0.618 | **0.694** | 0.565 |
| 0.15 | 1 | Singapore data | 0.388 | **0.535** | 0.429 |  | 0.15 | 1 | Singapore data | 0.588 | **0.688** | 0.588 |
| 0.05 | 2 | Singapore data | **0.9** | 0.882 | 0.865 |  | 0.05 | 2 | Singapore data | 0.859 | **0.888** | 0.853 |
| 0.1 | 2 | Singapore data | 0.812 | **0.871** | 0.829 |  | 0.1 | 2 | Singapore data | 0.724 | **0.812** | 0.788 |
| 0.15 | 2 | Singapore data | 0.806 | **0.865** | 0.847 |  | 0.15 | 2 | Singapore data | 0.665 | **0.806** | 0.788 |
| 0.05 | 3 | Singapore data | 0.929 | **0.953** | **0.953** |  | 0.05 | 3 | Singapore data | **0.947** | **0.947** | 0.929 |
| 0.1 | 3 | Singapore data | 0.853 | **0.906** | 0.888 |  | 0.1 | 3 | Singapore data | 0.824 | **0.9** | 0.865 |
| 0.15 | 3 | Singapore data | 0.918 | **0.924** | 0.882 |  | 0.15 | 3 | Singapore data | 0.865 | 0.888 | **0.9** |
| 0.05 | 1 | More Variance | 0.435 | 0.618 | **0.724** |  | 0.05 | 1 | More Variance | 0.747 | **0.8** | 0.741 |
| 0.1 | 1 | More Variance | 0.594 | **0.706** | 0.635 |  | 0.1 | 1 | More Variance | 0.629 | **0.735** | 0.594 |
| 0.15 | 1 | More Variance | 0.465 | **0.624** | 0.506 |  | 0.15 | 1 | More Variance | 0.582 | **0.724** | 0.576 |
| 0.05 | 2 | More Variance | 0.841 | 0.871 | **0.876** |  | 0.05 | 2 | More Variance | 0.847 | **0.882** | 0.859 |
| 0.1 | 2 | More Variance | 0.794 | **0.882** | 0.853 |  | 0.1 | 2 | More Variance | 0.735 | **0.818** | 0.794 |
| 0.15 | 2 | More Variance | 0.829 | **0.876** | 0.829 |  | 0.15 | 2 | More Variance | 0.653 | **0.8** | 0.782 |
| 0.05 | 3 | More Variance | 0.953 | **0.959** | 0.947 |  | 0.05 | 3 | More Variance | 0.935 | **0.953** | 0.935 |
| 0.1 | 3 | More Variance | 0.859 | 0.9 | **0.906** |  | 0.1 | 3 | More Variance | 0.847 | **0.888** | 0.847 |
| 0.15 | 3 | More Variance | 0.865 | 0.876 | **0.888** |  | 0.15 | 3 | More Variance | 0.847 | **0.888** | 0.882 |
| 0.05 | 1 | Most Variance | 0.594 | **0.729** | 0.712 |  | 0.05 | 1 | Most Variance | 0.771 | **0.818** | 0.741 |
| 0.1 | 1 | Most Variance | **0.512** | 0.465 | 0.365 |  | 0.1 | 1 | Most Variance | 0.659 | **0.753** | 0.629 |
| 0.15 | 1 | Most Variance | **0.335** | 0.3 | 0.218 |  | 0.15 | 1 | Most Variance | 0.635 | **0.747** | 0.647 |
| 0.05 | 2 | Most Variance | 0.847 | 0.876 | **0.9** |  | 0.05 | 2 | Most Variance | 0.847 | **0.876** | 0.853 |
| 0.1 | 2 | Most Variance | 0.806 | **0.853** | 0.812 |  | 0.1 | 2 | Most Variance | 0.765 | **0.829** | 0.8 |
| 0.15 | 2 | Most Variance | 0.806 | **0.853** | 0.835 |  | 0.15 | 2 | Most Variance | 0.665 | **0.806** | 0.776 |
| 0.05 | 3 | Most Variance | 0.929 | **0.947** | 0.935 |  | 0.05 | 3 | Most Variance | 0.941 | **0.947** | 0.935 |
| 0.1 | 3 | Most Variance | 0.871 | **0.935** | 0.9 |  | 0.1 | 3 | Most Variance | 0.865 | **0.9** | 0.865 |
| 0.15 | 3 | Most Variance | **0.906** | 0.894 | **0.906** |  | 0.15 | 3 | Most Variance | 0.853 | **0.876** | 0.912 |

#

# Table D in S1 File. Percentage accuracy of estimated angular reproduction number (Omega), temperature-independent reproduction number (ti-R_t_) and temperature-dependent reproduction number (td-R_t_) under simulations when transmission rate was underestimated by 4 times.

Percentage accuracy was calculated based on whether the significant transmissibility estimates and true Rt were both below 1 or above 1. The transmissibility estimate with the highest accuracy in each scenario was bolded.

| Noisy R |  |  |  |  |  |  | Smooth R |  |  |  |  |  |
| --- | --- | --- | --- | --- | --- | --- | --- | --- | --- | --- | --- | --- |
| Period | Amplitude | Temperature | Omega | td-Rt | ti-Rt |  | Period | Amplitude | Temperature | Omega | td-Rt | ti-Rt |
| 0.05 | 1 | Singapore data | 0.512 | 0.635 | **0.7** |  | 0.05 | 1 | Singapore data | 0.794 | **0.829** | 0.788 |
| 0.1 | 1 | Singapore data | 0.512 | **0.6** | **0.6** |  | 0.1 | 1 | Singapore data | 0.582 | **0.694** | 0.594 |
| 0.15 | 1 | Singapore data | 0.353 | **0.447** | 0.4 |  | 0.15 | 1 | Singapore data | 0.665 | **0.718** | 0.553 |
| 0.05 | 2 | Singapore data | 0.882 | **0.9** | **0.9** |  | 0.05 | 2 | Singapore data | 0.806 | **0.865** | 0.853 |
| 0.1 | 2 | Singapore data | 0.771 | **0.829** | 0.794 |  | 0.1 | 2 | Singapore data | 0.782 | **0.847** | 0.812 |
| 0.15 | 2 | Singapore data | 0.759 | **0.847** | 0.835 |  | 0.15 | 2 | Singapore data | 0.635 | **0.782** | 0.759 |
| 0.05 | 3 | Singapore data | 0.924 | **0.935** | 0.929 |  | 0.05 | 3 | Singapore data | 0.947 | **0.953** | 0.935 |
| 0.1 | 3 | Singapore data | 0.871 | **0.906** | 0.882 |  | 0.1 | 3 | Singapore data | 0.835 | **0.894** | 0.865 |
| 0.15 | 3 | Singapore data | 0.882 | **0.918** | **0.918** |  | 0.15 | 3 | Singapore data | 0.847 | 0.882 | **0.894** |
| 0.05 | 1 | More Variance | 0.347 | 0.518 | **0.682** |  | 0.05 | 1 | More Variance | 0.724 | **0.788** | 0.747 |
| 0.1 | 1 | More Variance | 0.553 | **0.682** | 0.618 |  | 0.1 | 1 | More Variance | 0.688 | **0.747** | 0.606 |
| 0.15 | 1 | More Variance | 0.476 | **0.524** | 0.382 |  | 0.15 | 1 | More Variance | 0.559 | **0.706** | 0.559 |
| 0.05 | 2 | More Variance | 0.859 | **0.894** | 0.865 |  | 0.05 | 2 | More Variance | 0.853 | **0.882** | 0.853 |
| 0.1 | 2 | More Variance | 0.829 | **0.871** | 0.824 |  | 0.1 | 2 | More Variance | 0.747 | **0.841** | 0.806 |
| 0.15 | 2 | More Variance | 0.782 | **0.853** | 0.824 |  | 0.15 | 2 | More Variance | 0.729 | **0.835** | 0.806 |
| 0.05 | 3 | More Variance | 0.935 | **0.953** | 0.935 |  | 0.05 | 3 | More Variance | 0.935 | **0.971** | 0.941 |
| 0.1 | 3 | More Variance | 0.853 | **0.9** | 0.876 |  | 0.1 | 3 | More Variance | 0.847 | **0.888** | 0.847 |
| 0.15 | 3 | More Variance | 0.894 | 0.906 | **0.924** |  | 0.15 | 3 | More Variance | 0.847 | 0.888 | **0.894** |
| 0.05 | 1 | Most Variance | 0.576 | 0.729 | **0.753** |  | 0.05 | 1 | Most Variance | 0.765 | **0.8** | 0.747 |
| 0.1 | 1 | Most Variance | 0.441 | **0.571** | 0.565 |  | 0.1 | 1 | Most Variance | 0.688 | **0.759** | 0.635 |
| 0.15 | 1 | Most Variance | 0.382 | **0.482** | 0.412 |  | 0.15 | 1 | Most Variance | 0.488 | **0.647** | 0.582 |
| 0.05 | 2 | Most Variance | **0.912** | **0.912** | 0.906 |  | 0.05 | 2 | Most Variance | 0.812 | 0.859 | **0.871** |
| 0.1 | 2 | Most Variance | 0.859 | **0.882** | 0.806 |  | 0.1 | 2 | Most Variance | 0.724 | **0.829** | 0.806 |
| 0.15 | 2 | Most Variance | 0.782 | 0.865 | 0.818 |  | 0.15 | 2 | Most Variance | 0.7 | **0.818** | **0.818** |
| 0.05 | 3 | Most Variance | 0.965 | **0.982** | 0.947 |  | 0.05 | 3 | Most Variance | 0.947 | **0.953** | 0.929 |
| 0.1 | 3 | Most Variance | 0.865 | **0.912** | 0.871 |  | 0.1 | 3 | Most Variance | 0.853 | **0.888** | 0.865 |
| 0.15 | 3 | Most Variance | 0.888 | **0.918** | 0.912 |  | 0.15 | 3 | Most Variance | 0.865 | 0.888 | **0.894** |

# Table E in S1 File. AUC-ROC values based on estimated reproduction numbers in simulation when transmission rate is overestimated and underestimated by 3 or 4 times respectively .

Each value describes the ability of the temperature-independent reproduction number (ti-Rt), angular reproduction number (Omega) and temperature-dependent reproduction number (td-Rt) to classify periods of transmission risk, where R_t_ > 1 is treated as the positive class (indicating epidemic growth) and R_t_$\leq$1 as the negative class. Higher AUC-ROC values indicate better classification performance, with the best value bolded. Each row corresponds to 1 simulation scenario with its respective temperature dataset and R_t_ dynamics.

| Transmission rate overestimated by 3 times | | | Transmission rate overestimated by 4 times | | | Transmission rate underestimated by 3 times | | | Transmission rate underestimated by 4 times | | |
| --- | --- | --- | --- | --- | --- | --- | --- | --- | --- | --- | --- |
| **td-Rt** | **Omega** | **ti-Rt** | **td-Rt** | **Omega** | **ti-Rt** | **td-Rt** | **Omega** | **ti-Rt** | **td-Rt** | **Omega** | **ti-Rt** |
| 0.995 | 0.997 | **0.999** | 0.995 | **0.997** | 0.999 | 0.995 | 0.997 | **0.999** | 0.995 | 0.997 | **0.999** |
| 0.986 | 0.984 | **0.993** | 0.986 | **0.984** | 0.993 | 0.986 | 0.984 | **0.993** | 0.986 | 0.984 | **0.993** |
| **0.974** | 0.971 | **0.969** | 0.974 | 0.971 | **0.969** | **0.974** | 0.971 | 0.969 | **0.974** | 0.971 | 0.969 |
| 0.995 | 0.971 | **1** | 0.995 | **0.971** | 1 | 0.995 | 0.971 | **1** | 0.995 | 0.971 | **1** |
| 0.989 | **0.996** | **0.996** | **0.989** | **0.996** | 0.996 | 0.989 | **0.996** | **0.996** | 0.989 | **0.996** | **0.996** |
| 0.992 | 0.995 | **0.996** | 0.992 | **0.995** | 0.996 | 0.992 | 0.995 | **0.996** | 0.992 | 0.995 | **0.996** |
| 0.997 | **0.999** | **0.999** | **0.997** | **0.999** | 0.999 | 0.997 | **0.999** | **0.999** | 0.997 | **0.999** | **0.999** |
| 0.994 | **0.996** | **0.996** | **0.994** | **0.996** | 0.996 | 0.994 | **0.996** | **0.996** | 0.994 | **0.996** | **0.996** |
| **1** | **1** | **0.999** | **1** | 1 | **0.999** | **1** | **1** | 0.999 | **1** | **1** | 0.999 |
| 0.955 | 0.964 | **0.974** | 0.955 | **0.964** | 0.974 | 0.955 | 0.964 | **0.974** | 0.955 | 0.964 | **0.974** |
| 0.988 | **0.992** | **0.992** | **0.988** | **0.992** | 0.992 | 0.988 | **0.992** | **0.992** | 0.988 | **0.992** | **0.992** |
| 0.98 | 0.986 | **0.989** | 0.98 | **0.986** | 0.989 | 0.98 | 0.986 | **0.989** | 0.98 | 0.986 | **0.989** |
| 0.991 | **0.995** | **0.994** | **0.991** | 0.995 | 0.994 | 0.991 | **0.995** | 0.994 | 0.991 | **0.995** | 0.994 |
| 0.995 | 0.995 | **0.996** | 0.995 | **0.995** | 0.996 | 0.995 | 0.995 | **0.996** | 0.995 | 0.995 | **0.996** |
| **0.995** | 0.994 | **0.994** | 0.995 | 0.994 | **0.994** | **0.995** | 0.994 | 0.994 | **0.995** | 0.994 | 0.994 |
| 0.995 | 0.996 | **0.999** | 0.995 | **0.996** | 0.999 | 0.995 | 0.996 | **0.999** | 0.995 | 0.996 | **0.999** |
| 0.995 | 0.995 | **0.996** | 0.995 | **0.995** | 0.996 | 0.995 | 0.995 | **0.996** | 0.995 | 0.995 | **0.996** |
| **0.993** | **0.993** | **0.993** | **0.993** | **0.993** | **0.993** | **0.993** | **0.993** | **0.993** | **0.993** | **0.993** | **0.993** |
| 0.974 | 0.975 | **0.978** | 0.974 | **0.975** | 0.978 | 0.974 | 0.975 | **0.978** | 0.974 | 0.975 | **0.978** |
| 0.981 | 0.988 | **0.993** | 0.981 | **0.988** | 0.993 | 0.981 | 0.988 | **0.993** | 0.981 | 0.988 | **0.993** |
| 0.987 | 0.983 | **0.989** | 0.987 | **0.983** | 0.989 | 0.987 | 0.983 | **0.989** | 0.987 | 0.983 | **0.989** |
| 0.996 | 0.997 | **0.999** | 0.996 | **0.997** | 0.999 | 0.996 | 0.997 | **0.999** | 0.996 | 0.997 | **0.999** |
| 0.997 | **0.999** | **0.999** | **0.997** | **0.999** | 0.999 | 0.997 | **0.999** | **0.999** | 0.997 | **0.999** | **0.999** |
| 0.994 | 0.996 | **0.997** | 0.994 | **0.996** | 0.997 | 0.994 | 0.996 | **0.997** | 0.994 | 0.996 | **0.997** |
| 0.998 | **1** | 0.998 | **0.998** | 1 | 0.998 | 0.998 | **1** | 0.998 | 0.998 | **1** | 0.998 |
| 0.996 | **0.999** | 0.998 | **0.996** | 0.999 | 0.998 | 0.996 | **0.999** | 0.998 | 0.996 | **0.999** | 0.998 |
| **1** | 0.999 | **0.998** | 1 | 0.999 | **0.998** | **1** | 0.999 | 0.998 | **1** | 0.999 | 0.998 |
| 0.988 | 0.991 | **0.992** | 0.988 | **0.991** | 0.992 | 0.988 | 0.991 | **0.992** | 0.988 | 0.991 | **0.992** |
| 0.966 | 0.972 | **0.975** | 0.966 | **0.972** | 0.975 | 0.966 | 0.972 | **0.975** | 0.966 | 0.972 | **0.975** |
| 0.979 | 0.976 | **0.98** | 0.979 | **0.976** | 0.98 | 0.979 | 0.976 | **0.98** | 0.979 | 0.976 | **0.98** |
| 0.988 | 0.989 | **0.992** | 0.988 | **0.989** | 0.992 | 0.988 | 0.989 | **0.992** | 0.988 | 0.989 | **0.992** |
| **0.988** | 0.994 | **0.996** | 0.988 | **0.994** | 0.996 | 0.988 | 0.994 | **0.996** | 0.988 | 0.994 | **0.996** |
| **0.998** | 0.996 | **0.997** | 0.998 | 0.996 | **0.997** | **0.998** | 0.996 | 0.997 | **0.998** | 0.996 | 0.997 |
| 0.992 | 0.998 | **1** | 0.992 | **0.998** | 1 | 0.992 | 0.998 | **1** | 0.992 | 0.998 | **1** |
| 0.99 | 0.991 | **0.993** | 0.99 | **0.991** | 0.993 | 0.99 | 0.991 | **0.993** | 0.99 | 0.991 | **0.993** |
| **0.996** | 0.993 | **0.982** | 0.996 | 0.993 | **0.982** | **0.996** | 0.993 | 0.982 | **0.996** | 0.993 | 0.982 |
| 0.978 | 0.979 | **0.988** | 0.978 | **0.979** | 0.988 | 0.978 | 0.979 | **0.988** | 0.978 | 0.979 | **0.988** |
| **0.996** | 0.991 | **0.994** | 0.996 | 0.991 | **0.994** | **0.996** | 0.991 | 0.994 | **0.996** | 0.991 | 0.994 |
| **0.966** | 0.962 | **0.965** | 0.966 | 0.962 | **0.965** | **0.966** | 0.962 | 0.965 | **0.966** | 0.962 | 0.965 |
| 0.999 | 0.998 | **1** | 0.999 | **0.998** | 1 | 0.999 | 0.998 | **1** | 0.999 | 0.998 | **1** |
| 0.993 | 0.996 | **0.998** | 0.993 | **0.996** | 0.998 | 0.993 | 0.996 | **0.998** | 0.993 | 0.996 | **0.998** |
| 0.993 | 0.994 | **0.995** | 0.993 | **0.994** | 0.995 | 0.993 | 0.994 | **0.995** | 0.993 | 0.994 | **0.995** |
| 0.992 | 0.998 | **1** | 0.992 | **0.998** | 1 | 0.992 | 0.998 | **1** | 0.992 | 0.998 | **1** |
| 0.997 | **0.998** | **0.998** | **0.997** | **0.998** | 0.998 | 0.997 | **0.998** | **0.998** | 0.997 | **0.998** | **0.998** |
| **0.999** | 0.998 | **0.999** | 0.999 | **0.998** | **0.999** | **0.999** | 0.998 | **0.999** | **0.999** | 0.998 | **0.999** |
| **0.987** | 0.986 | **0.986** | 0.987 | 0.986 | **0.986** | **0.987** | 0.986 | 0.986 | **0.987** | 0.986 | 0.986 |
| 0.984 | 0.986 | **0.987** | 0.984 | **0.986** | 0.987 | 0.984 | 0.986 | **0.987** | 0.984 | 0.986 | **0.987** |
| 0.954 | **0.956** | **0.956** | **0.954** | **0.956** | 0.956 | 0.954 | **0.956** | **0.956** | 0.954 | **0.956** | **0.956** |
| **0.993** | 0.991 | **0.991** | 0.993 | 0.991 | **0.991** | **0.993** | 0.991 | 0.991 | **0.993** | 0.991 | 0.991 |
| 0.994 | **0.996** | **0.996** | **0.994** | **0.996** | 0.996 | 0.994 | **0.996** | **0.996** | 0.994 | **0.996** | **0.996** |
| **0.998** | **0.998** | **0.998** | **0.998** | **0.998** | **0.998** | **0.998** | **0.998** | **0.998** | **0.998** | **0.998** | **0.998** |
| **1** | **1** | **0.999** | **1** | 1 | **0.999** | **1** | **1** | 0.999 | **1** | **1** | 0.999 |
| 0.996 | **0.997** | 0.996 | **0.996** | 0.997 | 0.996 | 0.996 | **0.997** | 0.996 | 0.996 | **0.997** | 0.996 |
| 0.995 | 0.992 | **0.996** | 0.995 | **0.992** | 0.996 | 0.995 | 0.992 | **0.996** | 0.995 | 0.992 | **0.996** |
