# Supplementary material for: Measuring real-time disease transmissibility with temperature-dependent generation intervals
Source: PLoS Comput Biol. 2026 Jan 21;22(1):e1013820. doi: 10.1371/journal.pcbi.1013820 (PMC12854435; doi:10.1371/journal.pcbi.1013820)
Supplement: Attachment — Submitted filename: review.docx [file pcbi.1013820.s002.docx]

The authors present a new way of taking temperature into account when calculating reproductive numbers for dengue, and explore in what scenarios different methods might be most accurate. This has important implications for better understanding dengue transmission in real-time, and the text is mostly very clear and detailed. I have several (mostly relatively minor) comments/questions below.

Abstract

Lines 22-25: It was unclear to me what this meant until I went and read the full text. In particular, “the agreement of sign around 1” is unclear without further context.

Introduction

Lines 51-53: It would be helpful to have a bit more detail here. What is the relationship between temperature and the incubation period (positive or negative, for example)? Do we know why?

Line 53: What species is this/are these?

Line 61: I’m unfamiliar with the WT method, so I’m not sure what the challenges are with using it to obtain real-time estimates. It would help to briefly explain this here.

Line 63: If EpiFilter uses future incidence data, wouldn’t it also not be applicable in real-time?

Methods

Line 85: Would it make sense to remove data from the time of the COVID-19 pandemic? Or do we not expect COVID-related nonpharmaceutical interventions to have had an impact on dengue transmission?

Line 89: If I understand correctly, this means that cases confirmed by rapid test are also included in the dataset? Or are these patients then also tested using PCR/ELISA to confirm?

Line 96: It says “temperature-independence” here, but I think it should be “temperature-dependence.”

Line 150: I’m not sure if η is ever defined.

Line 188-196: How sensitive is this method to the choice of delta?

Line 233: It’s not clear what is meant here by “four distinct groups.”

Results

Much of the results seems to repeat information from the Methods, which makes sense if the Methods will be at the end of the final manuscript. But if the Methods come before the Results, as in the current draft, I don’t think this is necessary. This information could be removed to make the Results much more concise.

Line 281: So when measured by AUC-ROC, ω outperforms ti-R_t_, but by percentage accuracy ti-R_t_ performs better? Why this discrepancy?

Lines 285-286: This doesn’t seem like such a large difference. Is it worth testing the methods again in scenarios where there is even more temperature variation? What about when there is less? Or is “more variation” referring more to the day-to-day change in temperature, rather than the range of temperatures covered (as shown in Figure 2)? If so, this should be better explained.

Lines 292-293: I’m a bit confused by this statement. In Table 2, it looks like ω rarely performs best, even when R_t_ is noisy.

Lines 295-297: I’m not sure I agree with this statement, based on Table 2. It looks like, in general, lower amplitude by itself does lead to overall lower accuracy across methods and datasets.

Lines 299-301: Why were these scenarios specifically chosen? What is meant by “not too extreme?”

Is there stochasticity involved in the methods to calculate ti-Rt/td-Rt/ω? In other words, would it be relevant to run the analyses several times, to ensure that your results are robust?

Line 303: What is meant by Fig. 3AJ, AA, etc.?

Line 307: Looking at Figure 2 and especially Supplementary Figure S4, the patterns in the simulated number of cases over time vary quite a bit by dataset. Are all of these somewhat realistic patterns to see for dengue outbreaks? I understand that you are testing the methods on synthetic data, but if the synthetic cases for a given scenario aren’t realistic, the results based on that scenario might also not be as relevant to real-world dengue outbreaks.

In Figure 2, the text in the rightmost 3 columns sometimes covers part of the lines. In the figure legend, can you also explain what the red lines in these columns are, and what the shaded areas represent? In the figure legend you mention blue labels, but I don’t see these on the figure.

Lines 341-342: What was the amount of agreement between ti-R_t_ and td-R_t_?

Lines 352-356: It is interesting that performance was so similar despite the fact that disagreement on the sign of R_t_ was not uncommon. Do you have any idea why this is? Would this change for longer forecast horizons?

The legends to the right of Figure 3 should be cleaned up. Some of this information is unnecessary or redundant.

Discussion

Overall, it would be good to see more interpretation of your results and advice for practical use throughout this section. For example, you find that td-R_t_ performs worse when there is a lot of noise in R_t_. What implications does this have for using these methods for real-world outbreaks? Do we know how noisy we expect R_t_ to be in reality? I also wonder if there might be any way to achieve more accurate results by combining the three metrics in some way – is this something that might be possible? Or, alternatively, is there some way to predict in real time which method might be most accurate for a given environment/scenario?

It would also be good to see some discussion of how your work could be applied in real-time analysis of transmission rates, since this seems to be the primary advantage of your work over that of Codeҫo et al. For example, sometimes case data from recent weeks are prone to error – how might this impact accuracy?

Lines 377-381: This information would go better in the introduction, so that readers understand why you have incorporated temperature in the way that you have.

Lines 386-388: Could you test this hypothesis? For example, you could run your analysis using synthetic temperature data with a wider range, as described for Northern Thailand.
