# Supplementary material for: Measuring real-time disease transmissibility with temperature-dependent generation intervals
Source: PLoS Comput Biol. 2026 Jan 21;22(1):e1013820. doi: 10.1371/journal.pcbi.1013820 (PMC12854435; doi:10.1371/journal.pcbi.1013820)
Supplement: Attachment — Submitted filename: Response to Reviewers.docx [file pcbi.1013820.s004.docx]

PCOMPBIOL-D-25-01284

Response to reviewers

Dear Dr Feilim Mac Gabhann and Dr Virginia Pitzer,

Thank you for the opportunity to submit a revised draft of the manuscript entitled “Measuring real-time disease transmissibility with temperature-dependent generation intervals” to *PLOS Computational Biology*. We appreciate the time and effort that you and the reviewers have dedicated to providing your valuable feedback on my manuscript. We are grateful to the reviewers for their insightful comments on my paper. We have been able to incorporate changes to reflect the suggestions provided by the reviewers and have tracked the changes within the manuscript. Here is a point-by-point response to the reviewers’ comments and concerns.

Comments from Editor

1. **Comment:** I share reviewer 1’s concern about the authors’ insufficient engagement with reporting delays/right truncation, and its implications on real-time applicability of the proposed method. The authors should consider updating their approach.

**Response:** We acknowledge the editor and reviewer 1’s concern about reporting delays and right truncation bias. Reviewer 1 commented on the omission of adjustment for delays in case reporting and recommended either to incorporate a right truncation correction or to explicitly discuss this limitation and its implications for real-time R_t_ estimation.

We would like to clarify that while our method did not explicitly address delays in case reporting, the Singapore dengue case data used in the manuscript was arranged based on date of illness onset rather than reporting date. Hence, the impact of such delays was likely minimal. Using dengue data by onset to mitigate the need for reporting delay adjustments is standard practice in the field (Ong et al., 2016; Shang et al., 2010). Nonetheless, if applied to case data arranged by reporting date, our methodology can be extended to adjust for right truncation bias with an additional nowcasting step adapted from methods like EpiNow2.

We have clarified that the dengue case data was arranged by date of illness onset in the Methods (lines 111-112):

“Daily dengue case data arranged by the date of illness onset from 2012 to 2024 was obtained from the Ministry of Health, Singapore.”

We have also revised the Discussion section to explicitly discuss this limitation in our methodology (lines 514-521):

“Furthermore, our approach did not explicitly account of reporting delays. In this study, the impact of such delays was likely minimal as we had access to the date of onset in the Singapore dengue case data (and not just the reporting date). As we also consider the serial interval distribution, which is the time between dates of onset, the incidence of onset times was the most appropriate data source.

Our methodology can be extended to account for reporting delays by incorporating a nowcasting step to adjust for right truncation bias. This could be done by estimating the expected number of cases that have occurred but have not yet been reported using historical reporting delay distributions and recent incidence patterns, as implemented in frameworks like EpiNow2.”

Furthermore, our framework remains highly applicable for real-time estimation by incorporating temperature to directly capture drivers of R_t_ beyond case counts and by using Bayesian recursive filtering to condition estimates on the full history of observed data. This makes it more stable than sliding-window approaches such as EpiEstim, which rely only on a fixed window of recent incidence. We have revised the Discussion to include these points (lines 444-450):

“Our framework is well-suited for real-time analysis as it incorporates temperature in the estimation of R_t_, allowing the method to capture drivers of transmission that are not directly observed from case data alone. Moreover, the Bayesian recursive filtering step in our framework improves the reliability of real-time estimates as they are conditioned on all past data. In contrast, real-time, sliding-window methods such as EpiEstim are unable to leverage the whole dataset and rely on only a fixed window of recent incidence data. These properties highlight the applicability of our framework for real-time analysis of transmission rates.”

2. **Comment:** Additionally, it would also be good for the authors to indicate whether the simulated data accounts for environmental factors other than temperature that impact transmission (for example, precipitation). This is perhaps related to some of the points raised by reviewer 2 about the representativeness of simulated data. Both reviewers suggest extending the analysis with temperature data from Northern Thailand.

Response: We thank the editor for their comment. Our simulated data focused on temperature as the primary environmental driver of transmission and did not explicitly incorporate other factors like precipitation. Nonetheless, the simulated cases remain representative of dengue outbreaks as they reproduce epidemic trajectories similar to those observed in real settings. In particular, we generated noisy R_t_ patterns to mimic the influence of external drivers that can cause short-term fluctuations in transmission, such as changes in human mobility or vector control. Additionally, we included scenarios where the generation interval distribution was misspecified, which serves as a proxy for unobserved factors like precipitation or vector control that also shape transmission dynamics. We have revised the Methods to clarify these points (lines 292-296):

“This mismatch between the true and assumed transmission dynamics allowed us to evaluate how sensitive the estimation framework is to misspecification of the generation interval, which may be driven by exogenous changes not captured by the model, such as period of intensified or relaxed vector control efforts which change the contact rate of humans and mosquitoes. The mismatch could also stem from changes in transmission driven by other environmental factors such as precipitation or humidity.”

In line with the reviewers’ suggestion, we have extended our analysis with temperature data from upper Northern Thailand, which has a wider temperature range. We have revised the Results, Methods and Discussion section to include the additional simulation results:

Methods:

“Furthermore, we used four different sets of temperature data in the different simulation scenarios, which were incorporated into the generation interval distribution of dengue when generating case data and estimating td-R_t_. The four datasets included daily temperature data in Singapore in 2012, daily temperature data in upper Northern Thailand (Chiang Mai, Lamphun, Lampang, Uttaradit, Phrae, Nan, Phayao, Chiang Rai, Mae Hong Son) in 2021, and two synthetic temperature datasets with more variance than Singapore’s temperature, which were normally distributed with a mean of 28 and standard deviation of 1.2 and 2 respectively. The Singapore temperature dataset had the smallest temperature range from 24.6°C – 30.0°C, followed by the two synthetic temperature datasets which ranged from 25.1°C – 31.2°C and 24.5°C – 32.7°C. The Northern Thailand temperature dataset had the widest temperature range of 18.0°C – 29.0°C. This allowed us to examine the performance of td-R_t_ under different temperature scenarios, specifically in settings with varying degrees of temperature fluctuations.”

Results:

“Overall, td-R_t_ had the best accuracy, outperforming $\Omega_{t}$ and ti-R_t_ in 54 out of 72 scenarios and tying with ti-R_t_ in 1 of the scenarios (Table 2). ti-R_t_ had the next best accuracy, which performed the best in 13 of the simulations (Table 2). The strong performance of td-R_t_ was corroborated by AUC-ROC values, which measured the classification ability of the R_t_ estimates. R_t_ > 1 was considered a positive class while R_t_ $\leq$ 1 was considered a negative class. td-R_t_ had the best AUC-ROC value in 56 out of 72 simulations (Fig. S3). In the other 16 simulations, the AUC-ROC value was only marginally smaller than the next best estimate (Fig. S3). Furthermore, $\Omega_{t}$ outperformed ti-R_t_ in 54 out of 72 simulations (Fig. S3).

We used different temperature datasets to define the generation interval distribution, which was then used to generate simulated dengue case counts. The Singapore temperature dataset exhibited the least variation, ranging from **24.6 °C** to 30.0 **°C**, while the Northern Thailand dataset had the most variation, ranging from 18.0 **°C** to 29.0 **°C** (Fig. S2). Based on the daily temperature of Northern Thailand, we estimated the generation time to have a mean of 16.7 days and variance of 2.49 days. Meanwhile, based on temperature in Singapore, the generation time was estimated to have a mean of 14.9 days and variance of 0.66 days. Despite these differences in temperature patterns, td-R_t_ consistently showed stronger accuracy across all temperature scenarios, ranging from 25.9% to 95.9% accuracy (Table 2). On the other hand, adding random noise to the underlying R_t_ of the simulated case counts potentially affected the performance of td-R_t_. Without random noise, td-R_t_ had the best accuracy in 33 out of 36 simulations with an accuracy of 70.0% to 96.5% (Table 2). With random noise, td-R_t_ had the best accuracy in 21 out of 36 simulations with an accuracy of 25.9% to 95.9% (Table 2). While $\Omega_{t}$ generally performed worse than td-R_t_ and ti-R_t_, it was comparatively less poor when the underlying R_t_ was noisy rather than smooth.”

“Generally, $\Omega_{t}$, td-R_t_ and ti-R_t_ estimated by dengue case counts generated using a true R_t_ with a lower amplitude had a lower absolute accuracy (Table 2). R_t_ estimated on data with a true R_t_ with an amplitude of 1 and period of 0.05 had an accuracy of 54.1% to 78.8%, whereas R_t_ estimated on data with a true R_t_ with a amplitude of 3 and period of 0.05 had an accuracy of 91.2% to 96.5% (Table 2). In Fig. 2, we narrowed our analysis down to scenarios where the amplitude of R_r_ was 2, which reflected realistic estimates of R_t_, and where underlying R_t_ was smooth to facilitate clearer visualisation. This allowed us to see how varying the temperature data set and the period of the true underlying R_t_ affected the accuracy of the transmissibility estimates. When temperature and underlying R_t_ fluctuates more, the difference in accuracy is largest between estimated td-R_t_ (75.9%) and the other 2 R_t_ estimates (58.2% & 56.5%) (Fig. 2I). When the temperature range was wide, as with the Northern Thailand temperature dataset, td-R_t_ had an accuracy of 73.5% while td-R_t_ and $\Omega_{t}$ had an accuracy of 62.9% and 57.6% respectively (Fig. 2K). In contrast, where temperature is more stable as in the Singapore dataset (Fig. 2A) or where the underlying R_t_ fluctuates less (Fig. 2A, 2D, 2G, 2J), the difference in accuracy between td-R_t_ and ti-R_t_ or $\Omega_{t}$ is less pronounced, though td-R_t_ still demonstrates higher accuracy. The full version of Fig. 2 with all simulation scenarios can be found in Fig. S4.”

Discussion:

“We showed that td-R_t_ would be particularly useful in regions which are more seasonal, such as upper Northern Thailand where daily temperatures varied from 18**°C in the cool season to 29°C in the hot season.** This is likely because the larger changes in temperature would result in larger changes in the generation interval. For example, the range of serial intervals in Northern Thailand is 60% larger than in Singapore, a difference that td-R_t_ is able to specifically account for, unlike ti-R_t_ or $\Omega$.”

Comments from Reviewer 1

1. **Comment:** Real-time estimation and right truncation bias

The manuscript claims to estimate Rt in real time. However, it does not appear to address right truncation bias due to delays in case reporting, which is a common issue in real-time epidemiological data. Without adjusting for this bias, the most recent Rt estimates are systematically underestimated, which can falsely suggest declining transmission. This undermines the use of the proposed real-time estimation framework in practical applications.

Given that statistical tools for nowcasting and correcting for right truncation are readily available (e.g., EpiNow2), the omission of such adjustments is a critical limitation. I strongly recommend that the authors either incorporate a right truncation correction or explicitly discuss this limitation and its implications for real-time Rt estimation.

**Response:** We thank the reviewer for their suggestion. We would like to clarify that while our method did not explicitly address delays in case reporting, the Singapore dengue case data used in the manuscript was arranged based on date of illness onset rather than reporting date. Hence, the impact of such delays was likely minimal. Nonetheless, if applied to case data arranged by reporting date, our methodology can be extended to adjust for right truncation bias with an additional nowcasting step adapted from methods like EpiNow2.

We have clarified that the dengue case data was arranged by date of illness onset in the Methods (lines 111-112):

“Daily dengue case data arranged by the date of illness onset from 2012 to 2024 was obtained from the Ministry of Health, Singapore.”

We have also revised the Discussion section to explicitly discuss this limitation in our methodology (lines 514-521):

“Furthermore, our approach did not explicitly account of reporting delays. In this study, the impact of such delays was likely minimal as the Singapore dengue case data was organized by date of illness onset rather than reporting date. Our methodology can be extended to account for reporting delays by incorporating a nowcasting step to adjust for right truncation bias. This could be done by estimating the expected number of cases that have occurred but have not yet been reported using historical reporting delay distributions and recent incidence patterns, as implemented in frameworks like EpiNow2.”

2. **Comment:** Scope of temperature variability in simulation scenarios

The differences between the temperature variation scenarios presented appear relatively small. To better demonstrate the benefits of including temperature in Rt estimation, I suggest incorporating an additional scenario with more pronounced seasonal variation, such as temperature data from northern Thailand.

**Response:** We thank the reviewer for their suggestion. We have included additional scenarios in our simulations using temperature data from upper Northern Thailand, consisting of the following provinces - Chiang Mai, Lamphun, Lampang, Uttaradit, Phrae, Nan, Phayao, Chiang Rai, Mae Hong Son. The daily temperature in Northern Thailand had the most seasonal variation across the temperature datasets, with a minimum of 18°C and a maximum of 29°C. The results from our simulation support our original conclusion that when temperature varies widely, the temperature-dependent reproduction number (td-R_t_) outperform the temperature-independent reproduction number (ti-R_t_) and angular reproduction number ($\Omega_{t}$) to a greater extent. This makes td-R_t_ particularly useful in regions with more seasonal variation. We have revised the Results, Methods and Discussion section to include the additional simulation results:

Methods:

“Furthermore, we used four different sets of temperature data in the different simulation scenarios, which were incorporated into the generation interval distribution of dengue when generating case data and estimating td-R_t_. The four datasets included daily temperature data in Singapore in 2012, daily temperature data in upper Northern Thailand (Chiang Mai, Lamphun, Lampang, Uttaradit, Phrae, Nan, Phayao, Chiang Rai, Mae Hong Son) in 2021, and two synthetic temperature datasets with more variance than Singapore’s temperature, which were normally distributed with a mean of 28 and standard deviation of 1.2 and 2 respectively. The Singapore temperature dataset had the smallest temperature range from 24.6°C – 30.0°C, followed by the two synthetic temperature datasets which ranged from 25.1°C – 31.2°C and 24.5°C – 32.7°C. The Northern Thailand temperature dataset had the widest temperature range of 18.0°C – 29.0°C. This allowed us to examine the performance of td-R_t_ under different temperature scenarios, specifically in settings with varying degrees of temperature fluctuations.”

Results:

“Overall, td-R_t_ had the best accuracy, outperforming $\Omega_{t}$ and ti-R_t_ in 54 out of 72 scenarios and tying with ti-R_t_ in 1 of the scenarios (Table 2). ti-R_t_ had the next best accuracy, which performed the best in 13 of the simulations (Table 2). The strong performance of td-R_t_ was corroborated by AUC-ROC values, which measured the classification ability of the R_t_ estimates. R_t_ > 1 was considered a positive class while R_t_ $\leq$ 1 was considered a negative class. td-R_t_ had the best AUC-ROC value in 56 out of 72 simulations (Fig. S3). In the other 16 simulations, the AUC-ROC value was only marginally smaller than the next best estimate (Fig. S3). Furthermore, $\Omega_{t}$ outperformed ti-R_t_ in 54 out of 72 simulations (Fig. S3).

We used different temperature datasets to define the generation interval distribution, which was then used to generate simulated dengue case counts. The Singapore temperature dataset exhibited the least variation, ranging from **24.6 °C** to 30.0 **°C**, while the Northern Thailand dataset had the most variation, ranging from 18.0 **°C** to 29.0 **°C** (Fig. S2). Despite these differences in temperature patterns, td-R_t_ consistently showed stronger accuracy across all temperature scenarios, ranging from 25.9% to 95.9% accuracy (Table 2). On the other hand, adding random noise to the underlying R_t_ of the simulated case counts potentially affected the performance of td-R_t_. Without random noise, td-R_t_ had the best accuracy in 33 out of 36 simulations with an accuracy of 70.0% to 96.5% (Table 2). With random noise, td-R_t_ had the best accuracy in 21 out of 36 simulations with an accuracy of 25.9% to 95.9% (Table 2). While $\Omega_{t}$ generally performed worse than td-R_t_ and ti-R_t_, it was comparatively less poor when the underlying R_t_ was noisy rather than smooth.”

“Generally, $\Omega_{t}$, td-R_t_ and ti-R_t_ estimated by dengue case counts generated a true R_t_ with a lower amplitude had a lower absolute accuracy (Table 2). R_t_ estimated on data with a true R_t_ with a amplitude of 1 and period of 0.05 had an accuracy of 54.1% to 78.8%, whereas R_t_ estimated on data with a true R_t_ with a amplitude of 3 and period of 0.05 had an accuracy of 91.2% to 96.5% (Table 2). In Fig. 2, we narrowed our analysis down to scenarios where the amplitude of R_r_ was 2, which reflected realistic estimates of R_t_, and where underlying R_t_ was smooth to facilitate clearer visualisation. This allowed us to see how varying the temperature data set and the period of the true underlying R_t_ affected the accuracy of the transmissibility estimates. When temperature and underlying R_t_ fluctuates more, the difference in accuracy is largest between estimated td-R_t_ (75.9%) and the other 2 R_t_ estimates (58.2% & 56.5%) (Fig. 2I). When the temperature range was wide, as with the Northern Thailand temperature dataset, td-R_t_ had an accuracy of 73.5% while td-R_t_ and $\Omega_{t}$ had an accuracy of 62.9% and 57.6% respectively (Fig. 2K). In contrast, where temperature is more stable as in the Singapore dataset (Fig. 2A) or where the underlying R_t_ fluctuates less (Fig. 2A, 2D, 2G, 2J), the difference in accuracy between td-R_t_ and ti-R_t_ or $\Omega_{t}$ is less pronounced, though td-R_t_ still demonstrates higher accuracy. The full version of Fig. 2 with all simulation scenarios can be found in Fig. S4.”

Discussion:

“We showed that td-R_t_ would be particularly useful in regions which are more seasonal, such as upper Northern Thailand where daily temperatures varied from 18**°C in the cool season to 29°C in the hot season.** This is likely because the larger changes in temperature would result in larger changes in the generation interval which td-R_t_ would be able to specifically account for, unlike ti-R_t_ or $\Omega$.”

3. **Comment:** Clarification on simulation design

The manuscript refers to 54 sets of simulated epidemic curves (line 222), but these are not introduced earlier. Please provide a clear description of how these simulation scenarios were generated and their purpose within the study design in the method section.

**Response:** We thank the reviewer for their comment. We acknowledge that how the simulation scenarios were generated, and their purpose could have been made clearer, earlier. We have revised the Methods section to include this description (lines 249-264).

“To investigate the effects of different underlying R_t_ values on the accuracy of the estimated R_t_, we varied the amplitude, period and smoothness of the underlying R_t_ values used to generate the case data. Smoothness was modified by adding random noise to the R_t_ values. Furthermore, we used four different sets of temperature data in the different simulation scenarios, which were incorporated into the generation interval distribution of dengue when generating case data and estimating td-R_t_. The four datasets included daily temperature data in Singapore in 2012, daily temperature data in upper Northern Thailand (Chiang Mai, Lamphun, Lampang, Uttaradit, Phrae, Nan, Phayao, Chiang Rai, Mae Hong Son) in 2021, and two synthetic temperature datasets with more variance than Singapore’s temperature, which were normally distributed with a mean of 28 and standard deviation of 1.2 and 2 respectively. The Northern Thailand temperature dataset had the widest temperature range of 18°C to 29°C. This allowed us to examine the performance of td- R_t_ under different temperature scenarios, specifically in settings with varying degrees of temperature fluctuations. A total of 72 simulated case datasets were generated by combining three amplitude levels of R_t_, three periodicities of R_t_, smooth versus non-smooth Rt dynamics, and four temperature datasets.”

4. **Comment:** Line 61: Consider clarifying that the real-time limitations of the Wallinga–Teunis method can be mitigated by incorporating nowcasting methods to account for reporting delays.

**Response:** We thank the reviewer for this suggestion. While nowcasting can correct reporting delays, it does not fully address the real-time limitations of the Wallinga–Teunis (WT) method, which arise from its use of future incidence data to estimate the reproduction number. The WT method probabilistically links each case to potential infectors using the generation interval distribution. From the infector-infectee pairs, it reconstructs the likely transmission network and epidemic trajectory. This leads to right censoring as there is missing information on the infectees at the end of the dataset where future cases have not been observed yet. This censoring issue would remain even with nowcasting. As such, WT is most often applied retrospectively, whereas real-time estimation typically relies on approaches such as EpiEstim by Cori et al. (2013) or EpiFilter Bayesian filtering methods used in the manuscript. We have revised the Introduction to clarify the real-time limitations of the WT method (lines 81-85):

“However, this was based on the WT method, which probabilistically links each case to potential infectors using the generation interval distribution. From these infector–infectee pairs, it reconstructs the likely transmission network and epidemic trajectory. This requires information on cases that occur after the time of inference, hence cannot be used to obtain real-time estimates and is more suited for retrospective analysis.”

5. **Comment:** Line 140: Please clarify whether a 35-day truncation period is sufficient to capture the generation interval across all temperature ranges considered in the analysis.

**Response:** We thank the reviewer for their question. The 35-day period is able to capture sufficient the generation interval across all temperature ranges. Specifically, across the range of temperatures in the analysis, the probability of a 35-day generation time is near zero. We have revised the Methods section to include this clarification (lines 171-173).

“The distribution satisfies $\sum_{u=1}^{U=\infty} w_{u} =1$ but was truncated to U=35 for computational efficiency, which captures over 99% of the probability mass across all temperature ranges used in the analysis.”

6. Figure 2: The legend refers to a "blue label," which does not appear clearly in the figure. Please revise or clarify.

**Response:** We thank the reviewer for their comment. We apologise for the mistake in the figure. The figure labels in Figure 2 have been revised to show the best-performing estimate in blue:

**Figure 2.** **Estimated angular reproduction number (Omega), estimated temperature-independent reproduction number (ti-Rt) and estimated temperature-dependent reproduction number (td-Rt) under simulation with varying temperature datasets, true underlying reproduction number (Rt) and cases.** Each row represents a set of simulation, which is run over 200 days. The values in the td-Rt, ti-Rt and Omega columns represent the percentage agreement by the threshold of 1 with the true Rt. (A), (B), (C): Singapore daily temperature; (D), (E), (F): Synthetic daily temperature data with more variation; (G), (H), (I): Synthetic daily temperature data with most variation. (J), (K), (L): Upper Northern Thailand daily temperature. The best-performing estimate is indicated by a blue label. In the td-Rt, ti-Rt, Omega columns, the red lines represent the estimated values, the black lines represent true values and shaded areas represent the 95% credible interval. Classification accuracy was calculated excluding the initial period of 24 days, indicated by the dashed line, to avoid edge effects.

7. Lines 218–219: Provide additional detail on the observed increase in variance. Articulate more details would help the reader better understand the impact.

**Response:** We thank the reviewer for their suggestion. We have revised the Methods to include more details about the different variances of the dataset (lines 254-262).

**“**The four datasets included daily temperature data in Singapore in 2012, daily temperature data in upper Northern Thailand (Chiang Mai, Lamphun, Lampang, Uttaradit, Phrae, Nan, Phayao, Chiang Rai, Mae Hong Son) in 2021, and two synthetic temperature datasets with more variance than Singapore’s temperature, which were normally distributed with a mean of 28 and standard deviation of 1.2 and 2 respectively. The Singapore temperature dataset had the smallest temperature range from 24.6°C – 30.0°C, followed by the two synthetic temperature datasets which ranged from 25.1°C – 31.2°C and 24.5°C – 32.7°C. The Northern Thailand temperature dataset had the widest temperature range of 18°C – 29°C. This allowed us to examine the performance of td-Rt under different temperature scenarios, specifically in settings with varying degrees of temperature fluctuations.”

Comments from Reviewer 2

1. **Comment:** Lines 22-25: It was unclear to me what this meant until I went and read the full text. In particular, “the agreement of sign around 1” is unclear without further context.

**Response:** We thank the reviewer for their comments. We acknowledge that the phrase “the agreement of sign around 1” may be unclear without context in the full text. We have revised the Abstract to clarify this phrasing (lines 20-27):

“Performance was evaluated by classification accuracy, defined by the proportion of instances where estimated R_t_ correctly identified whether the true R_t_ was above or below 1.

We found that td-R_t_ generally outperformed ti-R_t_ and $\Omega_{t}$ in classifying periods of epidemic growth. td-R_t_ achieved the highest classification accuracy in 42 of 54 simulation scenarios, with accuracy ranging from 37.1%–95.9%. td-R_t_ accuracy was highest in scenarios with greater temperature variability, surpassing other methods by up to 20%. With Singapore dengue case data, td-R_t_ and $\Omega_{t}$ signals showed 75% similarity, highlighting $\Omega_{t}$’s potential as a complementary measure that is less sensitive to model assumptions.”

2. **Comment:** Lines 51-53: It would be helpful to have a bit more detail here. What is the relationship between temperature and the incubation period (positive or negative, for example)? Do we know why?

**Response:** We thank the reviewer for their question. We have revised the Introduction to add more information on the relationship between temperature and incubation period (lines 70-74):

“In temperature-sensitive infectious diseases like dengue, biological processes underlying transmission are known to vary with temperature. Specifically, the extrinsic incubation period of dengue is biologically known to be temperature-dependent in the dengue vector species, *Aedes aegypti* (9). The extrinsic incubation period of dengue is longer at cooler temperatures, due to slower rates of viral replication within the vector (9,10).”

3. **Comment:** Line 53: What species is this/are these?

**Response:** We thank the reviewer for their question. The dengue vector species is *Aedes aegpyti* and *Aedes albopictus* we have included this in the description (lines 72-74, 126-127).

“Specifically, the extrinsic incubation period of dengue is biologically known to be temperature-dependent in the dengue vector species, *Aedes aegypti* and *Aedes albopictus* (9).”

“As *Aedes aegypti* is the primary and most efficient vector of dengue, the transmission stages were parameterized based on this species*.*”

4. **Comment:** Line 61: I’m unfamiliar with the WT method, so I’m not sure what the challenges are with using it to obtain real-time estimates. It would help to briefly explain this here.

**Response:** We thank the reviewer for their comment. We have revised the Introduction to clarify the limitations of the WT method (lines 81-85):

“However, this was based on the WT method, which probabilistically links each case to potential infectors using the generation interval distribution. From these infector–infectee pairs, it reconstructs the likely transmission network and epidemic trajectory. This requires information on cases that occur after the time of inference, hence cannot be used to obtain real-time estimates and is more suited for retrospective analysis.”

5. **Comment:** Line 63: If EpiFilter uses future incidence data, wouldn’t it also not be applicable in real-time?

**Response:** We thank the reviewer for their question. EpiFilter consists of a filtering step, which uses past incidence to generate real-time R_t_ estimates, and a smoothing step, which uses future incidence with respect to past estimates to refine the estimates retrospectively. Every estimate uses all available data but do not require future data. We have revised the Introduction to clarify that EpiFilter can be used in real-time applications (lines 87-90):

“EpiFilter is a recursive Bayesian smoother which uses forward filtering and backward smoothing to produce stable and improved R_t_ estimates by using all available data for every estimate (4). The filtering step uses only past incidence and can therefore provide reliable real-time estimates, while the smoothing step incorporates future incidence with respect to past estimates to refine those estimates as the later data had emerged.”

6. **Comment:** Line 85: Would it make sense to remove data from the time of the COVID-19 pandemic? Or do we not expect COVID-related nonpharmaceutical interventions to have had an impact on dengue transmission?

**Response:** We thank the reviewer for their question. While we expect COVID-related non-pharmaceutical interventions (NPIs) to impact dengue transmission, we chose not to exclude this portion of the data. Our goal was to estimate transmissibility and compare the estimates under real-world conditions, which include external factors such as NPIs. Excluding this data could reduce the generalisabiltiy of our findings.

Exogenous factors such as NPIs can influence estimates of R_t_ only via the generation interval, as R_t_ is defined by contemperaneous cases divided by past cases weighted by the generation interval. NPIs can only influence the generation interval by altering the time to transmission from either the human or the mosquito, as other components, such as the extrinsic and intrinsic incubation period, would be kept fixed or only varying based on temperature. However, we have done a sensitivity analysis simulation estimating R_t_ when human-to-mosquito and mosquito-to-human transmission rate were underestimated or overestimated, which could be a proxy for the potential impacts of COVID-NPIs on dengue transmission. We found that td-R_t_ similarly outperformed ti-R_t_ and $\Omega_{t}$ in such scenarios. This suggests the conclusion of our study remains the same whether data from COVID-19 is included or not.

We have revised the Methods to justify including the COVID-19 pandemic period of the dataset (lines 112-115):

“All available dengue case data, including the period overlapping with the COVID-19 pandemic, were retained to ensure that transmissibility estimates reflected real-world conditions, including potential effects of non-pharmaceutical interventions.”

7. **Comment:** Line 89: If I understand correctly, this means that cases confirmed by rapid test are also included in the dataset? Or are these patients then also tested using PCR/ELISA to confirm?

**Response:** We thank the reviewer for their question. Dengue cases confirmed by rapid tests are also included in the dataset. We have revised the Methods to clarify this point (lines 115-120):

“Dengue is a notifiable disease in Singapore under the Infectious Diseases Act which requires mandatory reporting of all laboratory-confirmed cases to the Ministry of Health (13). Confirmatory diagnostic testing for dengue is widely available in Singapore. Rapid diagnostic tests for dengue are commonly used in primary care, while additional tests such as PCR and ELISA are available in hospitals (14,15). Cases confirmed by rapid diagnostic tests are included in the dataset and are not always subsequently confirmed by PCR or ELISA.”

8. **Comment:** Line 96: It says “temperature-independence” here, but I think it should be “temperature-dependence.”

**Response:** We thank the reviewer for their comment and apologise for the typographical mistake. We have revised the Methods to correct this (lines 128-131):

“Temperature-dependence was incorporated into the extrinsic incubation period as the rate of viral replication within the mosquito vector is strongly influenced by ambient temperature, whereas the other stages in the transmission cycle (i.e. intrinsic incubation period, human-to-mosquito transmission and mosquito-to-human transmission) are less directly affected by temperature (9,15).”

9. **Comment:** Line 150: I’m not sure if η is ever defined.

**Response:** We thank the reviewer for their comment. We have revised the Methods to include the definition of η (lines 180-184).

“In EpiFilter, first, a state model is used to address the autocorrelation between reproduction numbers, characterised by the following equation:

$$R_{t}=R_{t-1}+(\eta\sqrt{R_{t-1}} )\epsilon_{t-1} (4)$$

$R_{t}$ is taken to be a hidden Markov state to be inferred, which dynamically depends on the previous state $R_{t-1}$ and a noise term $\epsilon_{t-1}\sim N(0,1)$. The noise term is scaled by a fraction, $\eta<1$, of $\sqrt{R_{t-1}}$."

10. **Comment:** Line 188-196: How sensitive is this method to the choice of delta?

**Response:** We thank the reviewer for their question. As shown in Parag et al. (2023), the estimation of angular reproduction was not sensitive choice of delta within the suggested range of twice to thrice of the mean generation time. The estimates start to vary and perform poorly when delta is very large (more than four times the mean generation time) as estimates become over-smoothed. We have clarified this point in the manuscript to note that our chosen delta value of twice the mean generation time falls within the range where the angular reproduction number is robust and reliable (lines 229-230):

“We set $\delta=24$ as it is heuristically recommended to be twice or thrice the mean generation interval time (6). Within this recommended range, the estimation of $\Omega_{t}$ is robust to the choice of $\delta$ (6).”

11. **Comment:** Line 233: It’s not clear what is meant here by “four distinct groups.”

**Response:** We thank the reviewer for their comment. We have revised the Methods to clarify what the four distinct groups of simulations were (lines 284-292):

“To assess the robustness of our transmissibility estimates, we evaluated the classification accuracy of the transmissibility estimates when the transmission rate, and in turn the generation interval, was misspecified. We generated four distinct groups of simulations, each with varying mismatches between the true and assumed transmission rate. Each group comprised of 54 simulated epidemic curves generated from R_t_ values with three amplitude levels, three periodicities, smooth versus non-smooth Rt dynamics, and three temperature datasets (Singapore temperature and two higher-variance synthetic temperature datasets). Two groups of simulations were based on a generation interval that underestimated transmission rate while two groups were based on a generation interval that overestimated transmission rate.”

12. **Comment:** Much of the results seems to repeat information from the Methods, which makes sense if the Methods will be at the end of the final manuscript. But if the Methods come before the Results, as in the current draft, I don’t think this is necessary. This information could be removed to make the Results much more concise.

**Response:** We thank the reviewer for their suggestion. We acknowledge the reviewer’s concern that the Results contain a reiteration of the Methods. This was written deliberately to guide and provide readers with immediate context about the results that follows and to ensure the results can be interpreted without frequent cross-referencing. While this may reduce the conciseness of the Results, we believe that it improves clarity and readability for the reader.

13. **Comment:** Line 281: So when measured by AUC-ROC, ω outperforms ti-Rt, but by percentage accuracy ti-Rt performs better? Why this discrepancy?

**Response:** We thank the reviewer for their question. This could be because AUC-ROC measures the model’s ability to separate the 2 classes - epidemic and controlled periods, at all thresholds of P(R_t_>1) and not only at P(R_t_>1) = 0.5. While $\Omega_{t}$ is better at separating the classes globally, td-R_t_ is better at classification at the P(R_t_>1) = 0.5 threshold. We note that policymakers may use differing probability thresholds for decision-making, and we chose 0.5 here as it provides a natural margin around the threshold of R_t_>1. We have revised the discussion to include this explanation (lines 490-496):

“When comparing the $\Omega_{t}$ and ti-R_t_, we noted that $\Omega_{t}$ generally achieved higher AUC-ROC values than ti-R_t_, whereas ti-R_t_ achieved a higher percentage accuracy than $\Omega_{t}$. AUC-ROC evaluates a model’s ability to discriminate between epidemic and controlled periods across all possible thresholds of P(R_t_>1). In contrast, percentage accuracy reflects a decision threshold at P(R_t_>1) = 0.5, which directly corresponds to the epidemiological threshold of R_t_=1. This suggests that while $\Omega_{t}$ may separate the two conditions more effectively, td-R_t_ aligns more closely with the practical decision boundary. While we chose a decision threshold of 0.5, policymakers may set lower or high cutoffs in practice.”

14. **Comment:** Lines 285-286: This doesn’t seem like such a large difference. Is it worth testing the methods again in scenarios where there is even more temperature variation? What about when there is less? Or is “more variation” referring more to the day-to-day change in temperature, rather than the range of temperatures covered (as shown in Figure 2)? If so, this should be better explained.

**Response:** We thank the reviewer for their suggestion. We have included additional scenarios in our simulations using temperature data from upper Northern Thailand, consisting of the following provinces - Chiang Mai, Lamphun, Lampang, Uttaradit, Phrae, Nan, Phayao, Chiang Rai, Mae Hong Son. The daily temperature in Northern Thailand had the most seasonal variation across the temperature datasets, with a minimum of 18°C and a maximum of 29°C. The results from our simulation support our original conclusion that when temperature varies widely, the temperature-dependent reproduction number (td-R_t_) outperform the temperature-independent reproduction number (ti-R_t_) and angular reproduction number ($\Omega_{t}$) to a greater extent. This makes td-R_t_ particularly useful in regions with more seasonal variation. We have revised the Results, Methods and Discussion section to include the additional simulation results:

Methods:

“Furthermore, we used four different sets of temperature data in the different simulation scenarios, which were incorporated into the generation interval distribution of dengue when generating case data and estimating td-R_t_. The four datasets included daily temperature data in Singapore in 2012, daily temperature data in upper Northern Thailand (Chiang Mai, Lamphun, Lampang, Uttaradit, Phrae, Nan, Phayao, Chiang Rai, Mae Hong Son) in 2021, and two synthetic temperature datasets with more variance than Singapore’s temperature, which were normally distributed with a mean of 28 and standard deviation of 1.2 and 2 respectively. The Singapore temperature dataset had the smallest temperature range from 24.6°C – 30.0°C, followed by the two synthetic temperature datasets which ranged from 25.1°C – 31.2°C and 24.5°C – 32.7°C. The Northern Thailand temperature dataset had the widest temperature range of 18.0°C – 29.0°C. This allowed us to examine the performance of td-R_t_ under different temperature scenarios, specifically in settings with varying degrees of temperature fluctuations.”

Results:

“Overall, td-R_t_ had the best accuracy, outperforming $\Omega_{t}$ and ti-R_t_ in 54 out of 72 scenarios and tying with ti-R_t_ in 1 of the scenarios (Table 2). ti-R_t_ had the next best accuracy, which performed the best in 13 of the simulations (Table 2). The strong performance of td-R_t_ was corroborated by AUC-ROC values, which measured the classification ability of the R_t_ estimates. R_t_ > 1 was considered a positive class while R_t_ $\leq$ 1 was considered a negative class. td-R_t_ had the best AUC-ROC value in 56 out of 72 simulations (Fig. S3). In the other 16 simulations, the AUC-ROC value was only marginally smaller than the next best estimate (Fig. S3). Furthermore, $\Omega_{t}$ outperformed ti-R_t_ in 54 out of 72 simulations (Fig. S3).

We used different temperature datasets to define the generation interval distribution, which was then used to generate simulated dengue case counts. The Singapore temperature dataset exhibited the least variation, ranging from **24.6 °C** to 30.0 **°C**, while the Northern Thailand dataset had the most variation, ranging from 18.0 **°C** to 29.0 **°C** (Fig. S2). Despite these differences in temperature patterns, td-R_t_ consistently showed stronger accuracy across all temperature scenarios, ranging from 25.9% to 95.9% accuracy (Table 2). On the other hand, adding random noise to the underlying R_t_ of the simulated case counts potentially affected the performance of td-R_t_. Without random noise, td-R_t_ had the best accuracy in 33 out of 36 simulations with an accuracy of 70.0% to 96.5% (Table 2). With random noise, td-R_t_ had the best accuracy in 21 out of 36 simulations with an accuracy of 25.9% to 95.9% (Table 2). While $\Omega_{t}$ generally performed worse than td-R_t_ and ti-R_t_, it was comparatively less poor when the underlying R_t_ was noisy rather than smooth.”

“Generally, $\Omega_{t}$, td-R_t_ and ti-R_t_ estimated by dengue case counts generated a true R_t_ with a lower amplitude had a lower absolute accuracy (Table 2). R_t_ estimated on data with a true R_t_ with a amplitude of 1 and period of 0.05 had an accuracy of 54.1% to 78.8%, whereas R_t_ estimated on data with a true R_t_ with a amplitude of 3 and period of 0.05 had an accuracy of 91.2% to 96.5% (Table 2). In Fig. 2, we narrowed our analysis down to scenarios where the amplitude of R_r_ was 2, which reflected realistic estimates of R_t_, and where underlying R_t_ was smooth to facilitate clearer visualisation. This allowed us to see how varying the temperature data set and the period of the true underlying R_t_ affected the accuracy of the transmissibility estimates. When temperature and underlying R_t_ fluctuates more, the difference in accuracy is largest between estimated td-R_t_ (75.9%) and the other 2 R_t_ estimates (58.2% & 56.5%) (Fig. 2I). When the temperature range was wide, as with the Northern Thailand temperature dataset, td-R_t_ had an accuracy of 73.5% while td-R_t_ and $\Omega_{t}$ had an accuracy of 62.9% and 57.6% respectively (Fig. 2K). In contrast, where temperature is more stable as in the Singapore dataset (Fig. 2A) or where the underlying R_t_ fluctuates less (Fig. 2A, 2D, 2G, 2J), the difference in accuracy between td-R_t_ and ti-R_t_ or $\Omega_{t}$ is less pronounced, though td-R_t_ still demonstrates higher accuracy. The full version of Fig. 2 with all simulation scenarios can be found in Fig. S4.”

Discussion:

“We showed that td-R_t_ would be particularly useful in regions which are more seasonal, such as upper Northern Thailand where daily temperatures varied from 18**°C in the cool season to 29°C in the hot season.** This is likely because the larger changes in temperature would result in larger changes in the generation interval which td-R_t_ would be able to specifically account for, unlike ti-R_t_ or $\Omega$.”

15. **Comment:** Lines 292-293: I’m a bit confused by this statement. In Table 2, it looks like ω rarely performs best, even when Rt is noisy.

**Response:** We thank the reviewer for their comment. We acknowledge that the previous statement might have been misleading. We have revised the statement in the Results to be more precise (lines 351-353):

“While $\Omega_{t}$ generally performed worse than td-R_t_ and ti-R_t_, it was comparatively less poor when the underlying R_t_ was noisy rather than smooth.”

16. **Comment:** Lines 295-297: I’m not sure I agree with this statement, based on Table 2. It looks like, in general, lower amplitude by itself does lead to overall lower accuracy across methods and datasets.

**Response:** We thank the reviewer for their observation and agree with this point. We have revised the statement to clarify that in general, lower amplitude of the underlying R_t_ does lead to overall lower accuracy (lines 354-357):

“Generally, $\Omega_{t}$, td-R_t_ and ti-R_t_ estimated by dengue case counts generated a true R_t_ with a lower amplitude had a lower absolute accuracy (Table 2). R_t_ estimated on data with a true R_t_ with a amplitude of 1 and period of 0.05 had an accuracy of 54.1% to 78.8%, whereas R_t_ estimated on data with a true R_t_ with a amplitude of 3 and period of 0.05 had an accuracy of 91.2% to 96.5% (Table 2).”

17. **Comment:** Lines 299-301: Why were these scenarios specifically chosen? What is meant by “not too extreme?”

Response: We thank the reviewer for their question. We selected scenarios with an amplitude of 2 because these are more realistic and better reflect the range of R_t_ typically estimated in real data. We also presented the smooth R_t_ scenarios to facilitate clearer visualization. We have revised the Results to clarify the justification for choosing to present these scenarios (lines 357-361):

“In Fig. 2, we narrowed our analysis down to scenarios where the amplitude of R_r_ was 2, which reflected realistic estimates of R_t_, and where underlying R_t_ was smooth to facilitate clearer visualisation. This allowed us to see how varying the temperature data set and the period of the true underlying R_t_ affected the accuracy of the transmissibility estimates.”

18. **Comment:** Is there stochasticity involved in the methods to calculate ti-Rt/td-Rt/ω? In other words, would it be relevant to run the analyses several times, to ensure that your results are robust?

Response: We thank the reviewer for their question. The methods we applied are deterministic given the incidence data and the specification of EpiFilter. The stochasticity is already accounted for within the EpiFilter framework, which captures uncertainty in R_t_ through its Bayesian recursive filtering and smoothing procedures. Specifically, at each time point, EpiFilter maintains a probability distribution over possible R_t_ values. As such, repeated runs will yield identical results. We have added text to the Methods to clarify this point in the manuscript (lines 205-208):

“EpiFilter is a deterministic algorithm and exact for a given prior and grid over the space of R_t,_ unlike sample-based approaches. Consequently, multiple runs at the same settings on the same data always produce the same posterior estimate.”

19. **Comment:** Line 303: What is meant by Fig. 3AJ, AA, etc.?

**Response:** We thank the reviewer for their question. We apologise for the mistake in labelling, and we have replaced them with the correct reference (lines 361-363):

“When temperature and underlying R_t_ fluctuates more, the difference in accuracy is largest between estimated td-R_t_ (75.9%) and the other 2 R_t_ estimates (58.2% & 56.5%) (Fig. 2I).”

20. **Comment:** Line 307: Looking at Figure 2 and especially Supplementary Figure S4, the patterns in the simulated number of cases over time vary quite a bit by dataset. Are all of these somewhat realistic patterns to see for dengue outbreaks? I understand that you are testing the methods on synthetic data, but if the synthetic cases for a given scenario aren’t realistic, the results based on that scenario might also not be as relevant to real-world dengue outbreaks.

Response: We thank the reviewer for their question. We simulated different patterns of case counts to reflect a range of outbreak dynamics that can plausibly occur. In large-scale outbreaks such as the 2024 dengue outbreak in Brazil, case counts can rise to several thousand per day. Meanwhile in smaller countries, dengue cases can increase from about 50 cases a day to 200 daily cases over a span of 3 months, as observed in the Singapore 2020 dengue outbreak. Our simulation included scenarios that span this spectrum but are still realistic, enabling our results to be relevant to real-world dengue outbreaks. We have revised the Results section to justify that our simulated cases were realistic (lines 264-271):

“The synthetic cases were simulated to reflect different plausible ranges of outbreak dynamics to ensure our results were relevant to real-world dengue epidemics. In large-scale outbreaks such as the 2024 dengue outbreak in Brazil, case counts can rise to several thousand per day. Meanwhile in smaller countries, dengue cases can increase from about 50 cases a day to 200 daily cases over a span of 3 months, as observed in the Singapore 2020 dengue outbreak. Our simulated case datasets were therefore constructed to capture this range of epidemic trajectories, allowing us to test model performance across diverse but realistic outbreak scenarios.”

21. **Comment:** In Figure 2, the text in the rightmost 3 columns sometimes covers part of the lines. In the figure legend, can you also explain what the red lines in these columns are, and what the shaded areas represent? In the figure legend you mention blue labels, but I don’t see these on the figure.

**Response:** We thank the reviewer for their suggestion. We have shifted the text labels in Figure 2 to not cover the lines and corrected the blue labels. We have also added an explanation for the red lines and shaded areas in the caption:

**Figure 2.** **Estimated angular reproduction number (Omega), estimated temperature-independent reproduction number (ti-Rt) and estimated temperature-dependent reproduction number (td-Rt) under simulation with varying temperature datasets, true underlying reproduction number (Rt) and cases.** Each row represents a set of simulation, which is run over 200 days. The values in the td-Rt, ti-Rt and Omega columns represent the percentage agreement by the threshold of 1 with the true Rt. (A), (B), (C): Singapore daily temperature; (D), (E), (F): Synthetic daily temperature data with more variation; (G), (H), (I): Synthetic daily temperature data with most variation. (J), (K), (L): Upper Northern Thailand daily temperature. The best-performing estimate is indicated by a blue label. In the td-Rt, ti-Rt, Omega columns, the black lines represent the true values, the red lines represent estimated values and shaded areas represent the 95% credible interval. Classification accuracy was calculated excluding the initial period of 24 days, indicated by the dashed line, to avoid edge effects.

22. **Comment:** Lines 341-342: What was the amount of agreement between ti-Rt and td-Rt?

**Response:** We thank the reviewer for their question. The overall percentage agreement between td-R_t_ and ti-R_t_ was 64.2%. We have added the overall agreement between td-R_t_ and ti-R_t_ in the Results (lines 409-413):

“The overall percentage agreement between td-R_t_ and ti-R_t_ was 64.2% (Table 3).

Table 3. Percentage agreement of angular reproduction number ($\boldsymbol{\Omega}$), temperature-dependent reproduction number (td-R_t_) and temperature-independent reproduction number (ti-R_t_).

|  | Both $\leq$ 1 or Both $>$ 1 (%) | Both not significant (%) | Total (%) |
| --- | --- | --- | --- |
| td-R_t_ **/** $\Omega_{t}$ | 19.0 | 56.0 | 75.0 |
| td-R_t_ / ti-R_t_ | 16.1 | 48.1 | 64.2 |
| ti-R_t_ **/** $\Omega_{t}$ | 15.5 | 32.5 | 48.0 |

”

23. **Comment:** Lines 352-356: It is interesting that performance was so similar despite the fact that disagreement on the sign of Rt was not uncommon. Do you have any idea why this is? Would this change for longer forecast horizons?

Response: We thank the reviewer for their question. As supported by Parag et al. (2024), the prediction performance is generally insensitive to the choice of generation time, as long as the R_t_ estimate and the corresponding total infectiousness used for estimation, $\Lambda_{t}$, are used together to generate predictions. As multi-step forecasts are iterated from the 1-step ahead forecast, we expect that the performance of longer forecast horizons will also have negligible differences. We have added this explanation to the Results section to explain the similarity in prediction performance (lines 423-427):

“This is because prediction performance is generally insensitive to the choice of generation time if the R_t_ estimate and the corresponding total infectiousness used for estimation, $\Lambda_{t}$, are used together to generate predictions. As multi-step forecasts are iterated from the 1-step ahead forecast, we expect that the performance of longer forecast horizons will also have negligible differences”

24. **Comment:** The legends to the right of Figure 3 should be cleaned up. Some of this information is unnecessary or redundant.

**Response:** We thank the reviewer for their comment. We have revised Figure 3 to remove the redundant legends:

Figure 3. (A) Dengue cases in Singapore, (B) angular reproduction number against temperature-dependent reproduction number (td-R_t_) and (C) angular reproduction number against temperature-independent reproduction number (ti-R_t_) estimated from Singapore dengue cases from 2012 to 2024. Grey shaded areas represent timepoints where the respective pair of R_t_ have concordant signals.

25. **Comment:** Discussion

Overall, it would be good to see more interpretation of your results and advice for practical use throughout this section. For example, you find that td-Rt performs worse when there is a lot of noise in Rt. What implications does this have for using these methods for real-world outbreaks? Do we know how noisy we expect Rt to be in reality? I also wonder if there might be any way to achieve more accurate results by combining the three metrics in some way – is this something that might be possible? Or, alternatively, is there some way to predict in real time which method might be most accurate for a given environment/scenario?

**Response:** We thank the reviewer for their suggestions. While we do not know how noisy R_t_ is in reality, we can expect R_t_ to be more noisy than smooth due to external factors which fluctuate in the short term, such as changes in human movement or vector control strategies like fogging which may confound transmissibility in the short term. However, we showed that even when R_t_ was noisy, td-R_t_ was still the most accurate overall. In real-life applications where decisions cannot rely on a single metric, the three metrics could be combined to achieve more accurate results, for example by categorizing periods of R_t_>1 and R_t_<=1 based on a majority vote across the three metrics. We have revised the Discussion to include these points (lines 473-480):

“While the true Rt in real-world settings is unknown, it is expected to fluctuate due to short-term factors such as human movement and vector control interventions like fogging. We found that in simulation scenarios where random external noise was introduced to the true underlying R_t_, $\Omega_{t}$ occasionally had a higher concordance with the true R_t_ than both td-R_t_ and ti-R_t_. However, td-R_t_ remained the most accurate metric overall. In practice, combining td-Rt, ti-R_t_, and $\Omega_{t}$ could leverage the strength of each metric and improve accuracy. One potential method would be to categorise periods of R_t_>1 and R_t_≤1 based on a majority vote across the three metrics. This approach could help produce more reliable estimates of transmissibility.”

26. **Comment:** It would also be good to see some discussion of how your work could be applied in real-time analysis of transmission rates, since this seems to be the primary advantage of your work over that of Codeҫo et al. For example, sometimes case data from recent weeks are prone to error – how might this impact accuracy?

Response: We thank the reviewer for their suggestion. We acknowledge that recent case data may be prone to error, which affects any real-time method estimating R_t_. Our framework remains robust by incorporating temperature to directly capture drivers of R_t_ beyond case counts and by using Bayesian recursive filtering to condition estimates on the full history of observed data. This makes it more stable than sliding-window approaches such as EpiEstim, which rely only on a fixed window of recent incidence. We have revised the Discussion to include these points (lines 444-450):

“Our framework is well-suited for real-time analysis as it incorporates temperature in the estimation of R_t_, allowing the method to capture drivers of transmission that are not directly observed from case data alone. Moreover, the Bayesian recursive filtering step in our framework improves the reliability of real-time estimates as they are conditioned on all past data. In contrast, real-time, sliding-window methods such as EpiEstim are unable to leverage the whole dataset and rely on only a fixed window of recent incidence data. These properties highlight the applicability of our framework for real-time analysis of transmission rates.”

27. **Comment:** Lines 377-381: This information would go better in the introduction, so that readers understand why you have incorporated temperature in the way that you have.

**Response:** We thank the reviewer for their suggestion. We have revised the Introduction to explain the role of temperature in dengue transmission (lines 72-74):

“Specifically, the extrinsic incubation period of dengue is biologically known to be temperature-dependent in the dengue vector species, *Aedes aegypti* (9). The extrinsic incubation period of dengue is longer at cooler temperatures, due to slower rates of viral replication within the vector (9,10).”

28. **Comment:** Lines 386-388: Could you test this hypothesis? For example, you could run your analysis using synthetic temperature data with a wider range, as described for Northern Thailand.

**Response:** We thank the reviewer for their suggestion. We have included additional scenarios in our simulations using temperature data from upper Northern Thailand, consisting of the following provinces - Chiang Mai, Lamphun, Lampang, Uttaradit, Phrae, Nan, Phayao, Chiang Rai, Mae Hong Son. The daily temperature in Northern Thailand had the most seasonal variation across the temperature datasets, with a minimum of 18°C and a maximum of 29°C. The results from our simulation support our original conclusion that when temperature varies widely, the temperature-dependent reproduction number (td-R_t_) outperform the temperature-independent reproduction number (ti-R_t_) and angular reproduction number ($\Omega_{t}$) to a greater extent. This makes td-R_t_ particularly useful in regions with more seasonal variation. We have revised the Results, Methods and Discussion section to include the additional simulation results:

Methods:

“Furthermore, we used four different sets of temperature data in the different simulation scenarios, which were incorporated into the generation interval distribution of dengue when generating case data and estimating td-R_t_. The four datasets included daily temperature data in Singapore in 2012, daily temperature data in upper Northern Thailand (Chiang Mai, Lamphun, Lampang, Uttaradit, Phrae, Nan, Phayao, Chiang Rai, Mae Hong Son) in 2021, and two synthetic temperature datasets with more variance than Singapore’s temperature, which were normally distributed with a mean of 28 and standard deviation of 1.2 and 2 respectively. The Singapore temperature dataset had the smallest temperature range from 24.6°C – 30.0°C, followed by the two synthetic temperature datasets which ranged from 25.1°C – 31.2°C and 24.5°C – 32.7°C. The Northern Thailand temperature dataset had the widest temperature range of 18.0°C – 29.0°C. This allowed us to examine the performance of td-R_t_ under different temperature scenarios, specifically in settings with varying degrees of temperature fluctuations.”

Results:

“Overall, td-R_t_ had the best accuracy, outperforming $\Omega_{t}$ and ti-R_t_ in 54 out of 72 scenarios and tying with ti-R_t_ in 1 of the scenarios (Table 2). ti-R_t_ had the next best accuracy, which performed the best in 13 of the simulations (Table 2). The strong performance of td-R_t_ was corroborated by AUC-ROC values, which measured the classification ability of the R_t_ estimates. R_t_ > 1 was considered a positive class while R_t_ $\leq$ 1 was considered a negative class. td-R_t_ had the best AUC-ROC value in 56 out of 72 simulations (Fig. S3). In the other 16 simulations, the AUC-ROC value was only marginally smaller than the next best estimate (Fig. S3). Furthermore, $\Omega_{t}$ outperformed ti-R_t_ in 54 out of 72 simulations (Fig. S3).

We used different temperature datasets to define the generation interval distribution, which was then used to generate simulated dengue case counts. The Singapore temperature dataset exhibited the least variation, ranging from **24.6 °C** to 30.0 **°C**, while the Northern Thailand dataset had the most variation, ranging from 18.0 **°C** to 29.0 **°C** (Fig. S2). Despite these differences in temperature patterns, td-R_t_ consistently showed stronger accuracy across all temperature scenarios, ranging from 25.9% to 95.9% accuracy (Table 2). On the other hand, adding random noise to the underlying R_t_ of the simulated case counts potentially affected the performance of td-R_t_. Without random noise, td-R_t_ had the best accuracy in 33 out of 36 simulations with an accuracy of 70.0% to 96.5% (Table 2). With random noise, td-R_t_ had the best accuracy in 21 out of 36 simulations with an accuracy of 25.9% to 95.9% (Table 2). While $\Omega_{t}$ generally performed worse than td-R_t_ and ti-R_t_, it was comparatively less poor when the underlying R_t_ was noisy rather than smooth.”

“Generally, $\Omega_{t}$, td-R_t_ and ti-R_t_ estimated by dengue case counts generated a true R_t_ with a lower amplitude had a lower absolute accuracy (Table 2). R_t_ estimated on data with a true R_t_ with a amplitude of 1 and period of 0.05 had an accuracy of 54.1% to 78.8%, whereas R_t_ estimated on data with a true R_t_ with a amplitude of 3 and period of 0.05 had an accuracy of 91.2% to 96.5% (Table 2). In Fig. 2, we narrowed our analysis down to scenarios where the amplitude of R_r_ was 2, which reflected realistic estimates of R_t_, and where underlying R_t_ was smooth to facilitate clearer visualisation. This allowed us to see how varying the temperature data set and the period of the true underlying R_t_ affected the accuracy of the transmissibility estimates. When temperature and underlying R_t_ fluctuates more, the difference in accuracy is largest between estimated td-R_t_ (75.9%) and the other 2 R_t_ estimates (58.2% & 56.5%) (Fig. 2I). When the temperature range was wide, as with the Northern Thailand temperature dataset, td-R_t_ had an accuracy of 73.5% while td-R_t_ and $\Omega_{t}$ had an accuracy of 62.9% and 57.6% respectively (Fig. 2K). In contrast, where temperature is more stable as in the Singapore dataset (Fig. 2A) or where the underlying R_t_ fluctuates less (Fig. 2A, 2D, 2G, 2J), the difference in accuracy between td-R_t_ and ti-R_t_ or $\Omega_{t}$ is less pronounced, though td-R_t_ still demonstrates higher accuracy. The full version of Fig. 2 with all simulation scenarios can be found in Fig. S4.”

Discussion:

“We showed that td-R_t_ would be particularly useful in regions which are more seasonal, such as upper Northern Thailand where daily temperatures varied from 18**°C in the cool season to 29°C in the hot season.** This is likely because the larger changes in temperature would result in larger changes in the generation interval which td-R_t_ would be able to specifically account for, unlike ti-R_t_ or $\Omega$.”
