# Supplementary material for: Measuring real-time disease transmissibility with temperature-dependent generation intervals
Source: PLoS Comput Biol. 2026 Jan 21;22(1):e1013820. doi: 10.1371/journal.pcbi.1013820 (PMC12854435; doi:10.1371/journal.pcbi.1013820)
Supplement: Attachment — Submitted filename: Response_to_Reviewers_auresp_2.docx [file pcbi.1013820.s005.docx]

PCOMPBIOL-D-25-01284

Response to reviewers

Dear Dr Feilim Mac Gabhann and Dr Virginia Pitzer,

Thank you for the opportunity to submit a revised draft of the manuscript entitled “Measuring real-time disease transmissibility with temperature-dependent generation intervals” to *PLOS Computational Biology*. We appreciate the time and effort that you and the reviewers have dedicated to providing your valuable feedback on my manuscript. We are grateful to the reviewers for their insightful comments on my paper. We have been able to incorporate changes to reflect the suggestions provided by the reviewers and have tracked the changes within the manuscript. Here is a point-by-point response to the reviewers’ comments and concerns.

Comments from Editor

1. **Comment**: *I am unsure how data being 'arranged' by onset date assures effect of reporting delays to be 'likely minimal', since data could be reported at a later date with the correct onset date, and thus an issue for real-time estimation. Perhaps further clarification on what the authors mean (or whether this assertion has been tested) and/or updates to relevant text in the manuscript could be useful.*

**Response:** We thank the editor for their comment. While cases are assigned to the correct date regardless of reporting delay, we acknowledge that late reporting can still lead to an underestimation of cases with recent onset. We have included an additional analysis to investigate the effect of underreporting on real-time td-R_t,_ ti-R_t_ and $\Omega_{t}$ estimates:

Methods

“We also investigated the effect of underreporting on the transmissibility estimates, where cases with recent onset may not have been reported yet. We assumed reporting delays to be lognormally distributed, with a mean reporting delay of 3 days and standard deviation of 3 days. This places most of the probability mass between 1-5 days, with a long tail to represent rare cases of long reporting delays up to 14 days. Each reported case in Singapore from 2022-2024 was assigned a reporting delay by adding a randomly drawn delay to the onset date to generate a simulated reporting date. Case counts with reporting dates beyond the latest timepoint were removed, which simulates right truncation and under-estimation of recent cases which have yet to be reported. We iteratively obtained the real-time estimates of td-R_t_, ti-R_t_ and $\Omega_{t}$ by re-running EpiFilter at each day using only data available up to that point, accounting for reporting delays.”

Results

“We also assessed how reporting delays and resulting underreporting influenced the real-time estimation of td-R_t_, ti-R_t_ and $\Omega_{t}$. Incorporating the reported delay distribution introduced a similar downward bias across all real-time estimates of td-R_t_, ti-R_t_ and $\Omega_{t}$ (Fig. S7). These results show that while underreporting leads to general underestimation in real-time inference, it affects each estimate in the same way. Nevertheless, td-R_t_ is likely to provide more accurate real-time inference than ti-R_t_, as supported by our simulation results.”

**Supplementary Figure S7 in S1 File. Real-time transmissibility estimates with simulated reporting delays.**

Each line shows the daily real-time estimates of temperature-dependent reproduction number (td-Rt), temperature-independent reproduction number (ti-Rt) and angular reproduction number (Omega) obtained using only data available up to that day, incorporating a lognormal reporting delay (mean = 3 days, SD = 3 days). The estimates capture how underreporting and right truncation influence transmissibility estimates over time, with all three metrics showing similar temporal patterns.

Discussion

“Furthermore, our approach did not explicitly account of reporting delays as we had access to the date of onset in the Singapore dengue case data (and not just the reporting date). As we also consider the serial interval distribution, which is the time between dates of onset, the incidence of onset times was the most appropriate data source. We found that in the presence of reporting delays, real-time transmissibility estimates may be underestimated. Our methodology can be extended to account for reporting delays by incorporating a nowcasting step to adjust for right truncation bias. This could be done by estimating the expected number of cases that have occurred but have not yet been reported using historical reporting delay distributions and recent incidence patterns, as implemented in frameworks like EpiNow2.”

2. **Comment:** *Additionally, the authors should consider more careful copy editing throughout to improve comprehension.*

**Response:** We thank the editor for their comment. We have revised the manuscript to improve readability and clarity.

Comments from Reviewer 2

1. **Comment:** Lines 87-92: Thank you for the clarification and additional details here. However, I still find this section confusing – you mention that EpiFilter can be used in real time, but then discuss a smoothing step that uses future incidence, which shouldn’t be available in real-time. In a real-time forecast, would only the filtering step be used?

**Response:** We thank the reviewer for this helpful clarification. In a real-time setting, only the filtering step would be used in the estimation of the latest time point. The smoothing step incorporates future information, thus is used in estimating all time points except the latest one. We have revised the text to clarify this distinction (line 88-92):

“The filtering step uses only past incidence and can therefore provide reliable real-time estimates, while the smoothing step incorporates future incidence with respect to past estimates to refine those estimates as the later data had emerged. As future information is required, the smoothing step is applied to refine estimates across all timepoints except the most recent one.”

2. **Comment:** Lines 290-296: Could you specify by how much the generation interval is over- or underestimated in each of the four groups?

**Response:** We thank the reviewer for their question. We have highlighted how overestimating or underestimating the transmission rate changes the length of the generation interval (lines 305-308):

“Overestimating transmission rate by 4 and 3 times shortens the generation interval by about 2 and 1 day respectively. Conversely, underestimating transmission rate by 4 and 3 times lengthens the generation interval by about 5 and 3 days respectively.”

3. **Comment:** Lines 264-271: Thank you for clarifying this. In addition to magnitude, are the simulations also realistic in terms of any observed seasonality in dengue cases? How many outbreaks are expected within a 200 day period of time?

**Response:** We thank the reviewer for their comment. We have explained how the seasonality of our simulations are realistic in the Methods (lines 270-276):

“The simulated cases generally display one major outbreak within the 200-day period. In these scenarios, case counts fluctuate over time but follow an overall increasing trend, which is consistent with the pattern typically observed in real dengue outbreaks. In other scenarios, the simulations show smaller fluctuations without a distinct peak, which may represent minor or well-controlled outbreaks. Some scenarios feature a single peak, resembling outbreaks commonly observed in Singapore, while others display multiple peaks, similar to patterns seen in larger or more seasonally variable countries such as Thailand or Brazil.”

4. **Comment:** Lines 419-427: I’m still a bit surprised about the similarity in error between these predictions. You say that predictions are insensitive to the generation time, but here, aren’t you estimating a reproductive number, then forming a corresponding forecast? Wouldn’t we expect the forecast generated from an Rt > 1 to be quite different than one generated with Rt < 1, as one would tend to produce an increase in cases and one a decrease? For the same reason, I would also tend to expect the difference in forecasts to compound over longer forecast horizons.

**Response:** We thank the reviewer for their question. In EpiFilter, predictions depend on the product of R_t_ and total infectiousness, $\Lambda_{t}$, which is calculated with generation time. When the generation time, and consequently $\Lambda_{t}$, is mis-specified, the inferred R_t_ adjusts in the opposite direction to the change in $\Lambda_{t}$. Hence, its product, which is the predicted incidence, remains almost unchanged even if its generation time or R_t_ differs. We have included this in the explanation to make it clearer (lines 443-448):

“This is because prediction performance is generally insensitive to the choice of generation time if the R_t_ estimate and the corresponding total infectiousness used for estimation, $\Lambda_{t}$, are used together to generate predictions (6). In EpiFilter, predictions depend on the product $R_{t}\Lambda_{t}$. When the generation time, and consequently $\Lambda_{t}$, is misspecified, the inferred $R_{t}$adjusts in the opposite direction. Hence, its product (predicted incidence) is almost unchanged even if the assumed generation time differs.”

5. **Comment:** Line 495: Instead of td-Rt, should this say ti_Rt?

**Response:** We thank the reviewer for their comment and apologise for the typographical mistake. We have revised the Discussion with the corrected term (lines 521-522):

“This suggests that while $\Omega_{t}$ may separate the two conditions more effectively, ti-R_t_ aligns more closely with the practical decision boundary.”

6. **Comment:** Lines 444-450: While it makes sense that, by taking temperature into account, you are not relying only on case counts, I still wonder to what extent underreporting and underestimation of cases in current and recent weeks might affect real-time estimation of Rt. Is this something you could test – for example, by misspecifying the number of cases with symptom onset in recent weeks to represent underreporting, then testing whether you get the same results?

**Response:** We thank the reviewer for their comment. While cases are assigned to the correct date regardless of reporting delay, we acknowledge that late reporting can still lead to an underestimation of cases with recent onset. We have included an additional analysis to investigate the effect of underreporting on real-time td-R_t,_ ti-R_t_ and $\Omega_{t}$ estimates:

Methods

“We also investigated the effect of underreporting on the transmissibility estimates, where cases with recent onset may not have been reported yet. We assumed reporting delays to be lognormally distributed, with a mean reporting delay of 3 days and standard deviation of 3 days. This places most of the probability mass between 1-5 days, with a long tail to represent rare cases of long reporting delays up to 14 days. Each reported case in Singapore from 2022-2024 was assigned a reporting delay by adding a randomly drawn delay to the onset date to generate a simulated reporting date. Case counts with reporting dates beyond the latest timepoint were removed, which simulates right truncation and under-estimation of recent cases which have yet to be reported. We iteratively obtained the real-time estimates of td-R_t_, ti-R_t_ and $\Omega_{t}$ by re-running EpiFilter at each day using only data available up to that point, accounting for reporting delays.”

Results

“We also assessed how reporting delays and resulting underreporting influenced the real-time estimation of td-R_t_, ti-R_t_ and $\Omega_{t}$. Incorporating the reported delay distribution introduced a similar downward bias across all real-time estimates of td-R_t_, ti-R_t_ and $\Omega_{t}$ (Fig. S7). These results show that while underreporting leads to general underestimation in real-time inference, it affects each estimate in the same way. Nevertheless, td-R_t_ is likely to provide more accurate real-time inference than ti-R_t_, as supported by our simulation results.”

**Supplementary Figure S7 in S1 File. Real-time transmissibility estimates with simulated reporting delays.**

Each line shows the daily real-time estimates of temperature-dependent reproduction number (td-Rt), temperature-independent reproduction number (ti-Rt) and angular reproduction number (Omega) obtained using only data available up to that day, incorporating a lognormal reporting delay (mean = 3 days, SD = 3 days). The estimates capture how underreporting and right truncation influence transmissibility estimates over time, with all three metrics showing similar temporal patterns.

Discussion

“Furthermore, our approach did not explicitly account of reporting delays as we had access to the date of onset in the Singapore dengue case data (and not just the reporting date). As we also consider the serial interval distribution, which is the time between dates of onset, the incidence of onset times was the most appropriate data source. We found that in the presence of reporting delays, real-time transmissibility estimates may be underestimated. Our methodology can be extended to account for reporting delays by incorporating a nowcasting step to adjust for right truncation bias. This could be done by estimating the expected number of cases that have occurred but have not yet been reported using historical reporting delay distributions and recent incidence patterns, as implemented in frameworks like EpiNow2.”
